# Liver Transplantation for Fibrolamellar Carcinoma: National Registry Analysis and Reported Outcomes

**DOI:** 10.3390/cancers18203335

**Published:** 2026-10-15

**Authors:** Piyush Gupta, Dimitrios Moris, Caitlin Mea, David Walls, Shekhar Gogna, Oswaldo Aguirre, Ioannis A. Ziogas, Brian M. Nguyen

**Affiliations:** 1 MedStar Georgetown Transplant Institute, MedStar Georgetown University Hospital, 3800 Reservoir Road NW, Washington, DC 20007, USAdavid.walls@medstar.net (D.W.); oswaldo.aguirre@gunet.georgetown.edu (O.A.);; 2 Department of Surgery, Wake Forest University School of Medicine, Winston-Salem, NC 27157, USA; 3 Department of Surgery, University of Colorado Anschutz Medical Campus, Aurora, CO 80045, USA

**Keywords:** fibrolamellar carcinoma, liver transplantation, recurrence, transplant oncology, scientific registry of transplant recipients, competing risks, prediction model

## Abstract

**Background/Objectives:** Liver transplantation may provide prolonged survival in fibrolamellar carcinoma (FLC), but the frequency and timing of post-transplant recurrence, and the basis for patient selection, remain poorly defined. We described the course from listing through long-term follow-up and tested whether routine pretransplant measurements distinguished the recurrence risk. **Methods:** Scientific Registry of Transplant Recipients data identified separate listing and first-transplant cohorts during 2002–2025. Competing-risk analyses assessed waiting-list outcomes and reported recurrence of the pretransplant tumour. Exploratory recurrence models were internally validated with 500 patient bootstrap samples, with performance assessed only in omitted recipients. Recorded explant tumour extent was examined in the registry’s liver explant file. **Results:** This study included 61 listed candidates and 51 first-transplant recipients; 42 recipients had a pathology-supported discharge diagnosis of FLC. One-year incidence of transplantation was 76.5% (95% CI 65.9–86.6). After a median potential follow-up of 8.1 years, five-year post-transplant survival was 63.3% (95% CI 46.4–76.2). Seventeen recurrences were reported and the three-year cumulative incidence was 26.1% (95% CI 13.9–39.1). Malignancy was the recorded primary cause in 13 of 23 deaths. Laboratory-component and compact clinical models had mean three-year validation areas under the curve of 0.571 and 0.507, and neither reduced prediction error relative to its matched cohort-average prediction. **Conclusions:** Transplantation was followed by prolonged survival in selected patients with FLC, although reported recurrence was common and sometimes late. Routine pretransplant variables did not identify reproducible recurrence-risk groups. With 51 recipients and 17 recurrences, these estimates are imprecise and the prediction analyses are exploratory; selection should remain multidisciplinary while tumour extent, treatment response and biological markers are evaluated prospectively.

## Simple Summary

Fibrolamellar carcinoma is a rare liver cancer that mostly affects teenagers and young adults who are otherwise healthy. When the tumour cannot be removed by an operation, a liver transplant is sometimes offered. Doctors do not know how often the cancer returns afterwards, or how to tell in advance which patients are most at risk. We studied every patient in the United States national transplant registry whose records identified fibrolamellar carcinoma, over more than twenty years. Most patients who were accepted onto the waiting list did receive a transplant, and many lived for years afterwards. Cancer did return in a substantial minority, sometimes more than three years later. Routine blood tests and clinical measurements taken before the transplant did not reliably predict who would have a recurrence. Because the number of patients is small, these results are exploratory and should guide future study rather than patient selection.

## 1. Introduction

Fibrolamellar carcinoma (FLC) usually affects young patients without cirrhosis, in whom a large hepatic tumour may be unresectable despite preserved liver function. Craig and colleagues first systematically delineated it as a tumour of adolescents and young adults with distinctive clinicopathological features [1], and population-based analyses have since confirmed that its demographic profile and natural history differ from those of conventional hepatocellular carcinoma (HCC) [2,3]. Its characteristic morphology and recurrent DNAJB1::PRKACA fusion distinguish it from conventional HCC [4,5], and molecular confirmation of the fusion now supports the clinical diagnosis in the appropriate morphological setting [6].

Complete resection remains the principal treatment when feasible; single-institution series and systematic review report the longest survival after resection [7,8], and the most recent synthesis concludes that resection is currently the only curative treatment available [9]. Mayo and colleagues found that surgically managed patients with FLC survived a median of 75 months against 43 months for conventional HCC, despite presenting more often with regional disease [10]. For unresectable disease confined to the liver, transplantation offers complete hepatic tumour removal, but the likelihood of lasting tumour control is central to selection. Pinna and colleagues reported long-term survivors after aggressive surgical treatment of advanced disease, with transplantation reserved for tumours not amenable to partial hepatectomy [11]. Recurrence followed transplantation in 9 of 13 of their recipients, and the authors declined to compare survival across modalities because the indications differed. In the Birmingham experience, in which transplantation was reserved for unresectable tumours, three-year survival was 76% after transplantation against 100% after resection [12]. The FLC Consortium associated prognosis with disease extent and surgical treatment [13]. Atienza and colleagues reported 63 United Network for Organ Sharing recipients, with survival of 96%, 80% and 48% at one, three and five years [14]. FLC is now discussed alongside the other accepted rare oncological indications for transplantation [15,16], and the 2026 multidisciplinary guideline strongly recommends considering transplantation for unresectable, liver-confined FLC irrespective of Milan criteria [17]. Kassahun’s review found long-term outcomes after resection favourable when it formed part of aggressive therapy, while median survival in unresectable disease remained under 12 months [18]. Among the 94 patients reported by Kaseb and colleagues, early tumour stage and resection including metastasectomy were associated with longer overall survival, but sex was the only variable associated with recurrence-free survival [19].

Outcomes after acceptance for listing and the timing of recurrence are therefore less well characterised than recipient survival. This study examined the course from listing through long-term follow-up, reconstructed reported recurrence after transplantation, and tested whether routinely collected pretransplant measurements could distinguish recurrence risk. The registry’s liver explant file, which records pathological findings from the explanted liver, allowed exploratory assessment of recorded tumour extent. Because FLC is rare, the analysis was designed as a descriptive and hypothesis-generating account of national practice rather than as a test of transplant benefit or a source of selection criteria.

## 2. Materials and Methods

### 2.1. Data Sources and Study Population

This study used data from the Scientific Registry of Transplant Recipients (SRTR). The SRTR data system includes data on all donor, wait-listed candidates, and transplant recipients in the US, submitted by the members of the Organ Procurement and Transplantation Network (OPTN). The Health Resources and Services Administration (HRSA), U.S. Department of Health and Human Services, provides oversight to the activities of the OPTN and SRTR contractors. This retrospective study used SRTR standard analysis files, release 2606. SRTR compiles the data submitted by transplant centers and organ procurement organisations for every transplant candidate and recipient in the United States, and supplements outcome ascertainment with additional sources [20]. All analyses in this study, including the explant analysis in Section 2.4, draw on this single registry. Candidate, transplant, follow-up, exception, and status-history records were linked using supplied identifiers. Entry was restricted to 27 February 2002 through 31 December 2025, with follow-up censored on 31 December 2025. Reporting followed STROBE and RECORD guidance [21,22], and the prediction analyses follow TRIPOD reporting items for model development with internal validation [23]. Detailed definitions and sensitivity analyses are provided in the Supplementary Materials.

FLC was identified by diagnosis code 4402 or explicit fibrolamellar wording, including reviewed spelling variants. Records naming an incompatible tumour were excluded; generic HCC coding accompanying an explicit FLC subtype was retained. REC_DGN2, the center-reported pathology-confirmed liver diagnosis at hospital discharge, provided separate pathological support for the transplant diagnosis. This field does not represent central specimen review.

Listing and recipient cohorts were constructed separately, with one observation per patient. Listing began at the earliest eligible native-liver registration across available history; never-waitlisted living-donor recipients were excluded. Recipients entered at their first observed liver transplant with qualifying FLC evidence and no previous-liver-transplant flag. The earliest eligible record was selected before applying the entry window. All ages and liver-containing multiorgan procedures were eligible. Recipient-only diagnosis information could not establish listing-cohort membership. The registry does not record the clinical criteria on which centers accepted these candidates for listing; the listing cohort therefore comprises every candidate registered with a qualifying FLC diagnosis, whatever the listing center’s selection practice.

### 2.2. Clinical Outcomes

The waiting-list endpoint was the first exit from the continuous listing episode: transplantation, death or removal for clinical deterioration, or other removal. Concurrent registrations were combined. Administrative transfers continued the episode only when another registration documented same-day or next-day continuation. Definitive removal ended the episode, even if the patient was later relisted; linked transplantation took precedence over same-day removal. Overall survival from listing continued through removal, relisting, and transplantation where subsequent observations were available. Recipient survival began at the first transplant. All-cause index-graft loss comprised death, recorded graft failure or retransplantation.

Death dates were reconciled across the registry record, center follow-up, and Social Security sources. Concordant clinical dates took priority, with Social Security dates used when clinical dates were unavailable. Survivors were censored at their last observed vital follow-up or the administrative horizon. Primary cause of death came from the registry record corresponding to the selected death date and was checked against same-date deceased-status follow-up. Malignancy-coded death and graft failure attributed to recurrent disease were retained separately; neither establishes histologically confirmed recurrent FLC.

Reported recurrence was the first recurrence of a pretransplant tumour after the index transplant. A dated malignancy record had to link to its parent index-graft follow-up form recording both malignancy and recurrence of a pretransplant tumour. Death without reported recurrence was a competing event and was handled as such throughout [24]. Non-event follow-up ended at the last explicit negative oncology assessment, rather than at a later vital-status contact with unknown oncology status. A vital-status contact establishes that the recipient was alive but not whether recurrence had been looked for, so counting that interval as recurrence-free could understate the incidence; ending follow-up at the last explicit negative assessment counts only the time in which recurrence status was recorded. Two recipients without post-transplant oncology ascertainment were censored at day zero. A negative form after recurrence did not reverse the event or establish an earlier recurrence-free interval. Sensitivities used the first positive report date, alternative graft scope, stricter date checks, generic vital-status censoring, and the pathology-supported diagnosis subgroup. A further sensitivity narrowed the endpoint to recurrence coded to the liver or biliary tract, treating other or unspecified sites as a competing event.

### 2.3. Pretransplant Characteristics and Recurrence Prediction

Clinical descriptions included age, sex, donor and transplant type, and laboratory model for end-stage liver disease (MELD) or pediatric end-stage liver disease (PELD) scores. Baseline tables used initial candidate scores for the listing cohort and last available candidate scores for recipients. Exception applications were distinguished from approved effective priority periods within the first listing episode. HCC-labeled tumour, biopsy, and treatment fields were examined for their definitions and observation dates.

Exploratory prediction analyses were added sequentially after the descriptive analysis. Each extension specified its predictors and fixed penalties after assessing availability and before examining the new predictor-outcome associations. An age-and-sex benchmark used Fine–Gray and ridge-penalized cause-specific models. The laboratory-component model included bilirubin, international normalized ratio, creatinine, albumin, sodium, hospitalization immediately before transplantation and associated-registration waiting time, with logarithmic transformations specified in the Supplementary Materials. The compact clinical model included laboratory MELD, albumin, hospitalization, and waiting time; PELD recipients were excluded. Laboratories came from the latest status record beginning strictly before transplantation, whose date was not assumed to be the blood-draw date. Alanine aminotransferase (ALT) was studied separately among recipients with an admissible historical value, using log2 ALT and continuous transplant year, with year-only and cohort-average benchmarks. A descriptive ALT hazard ratio was adjusted for year. Structured tumour assessments (alpha-fetoprotein, tumour number, and macrovascular and extrahepatic status) are collected on exception application forms and were available for only 14 of the 61 listed candidates (Table S9) and 13 of the 51 recipients (Table S12). They were therefore described but neither entered as predictors nor imputed, because imputation would have supplied the value for about three-quarters of the recipients. Associated-registration waiting time is an interval to an available donor and not a tumour-observation interval; in a cohort with a short median wait it partly reflects donor availability rather than disease biology.

Clinical models used ridge-penalized cause-specific recurrence regression and a common competing-death hazard. Penalties were fixed rather than tuned to apparent performance. Bounded exponential integration of the fitted hazards was used to convert them into cumulative-incidence predictions for the expanded models; the Supplementary Materials report the numerical correction and failures of the original product-limit calculation. Internal validation used 500 patient bootstrap samples, repeating imputation, transformations, standardization and fitting within each training sample [25]. Performance was evaluated only in recipients omitted from that sample. Mean validation estimates were calculated for censoring-weighted three-year area under the curve (AUC) and Brier score [26,27]. AUC measured discrimination, while a lower Brier score indicated less prediction error; both were interpreted against the matched training-sample cohort-average prediction [27]. Relative higher/lower assignments based on training-sample median risk were examined for reproducibility; no transplant eligibility cutoff was derived. The number of recurrence events available was far below that required to develop a stable clinical prediction model [28]; so, these analyses are interpreted only as a test of whether routine variables carry any reproducible recurrence signal.

### 2.4. Exploratory Explant Analysis

Structured explant data were assessed in the registry’s liver explant file, which records pathological findings from the explanted liver and covers records through 31 March 2026. Eligibility required code 4402, a first observed liver transplant, no previous-transplant flag and the same study entry window. Supplied identifiers linked index-transplant explant and oncology forms. The explant file carries its own encrypted transplant identifier, so records were linked within that file rather than merged with the primary analysis files. The explant analysis is therefore a second pass over the same registry rather than an independent population, and the two were not pooled. Before recurrence comparison, extent was grouped as any recorded vascular invasion, nodal involvement or extrahepatic spread; explicit absence of all three; or incomplete classification. Available follow-up and three-year outcomes distinguished reported recurrence, a recorded negative oncology assessment covering the horizon, and unknown status. Explant findings were postoperative observations.

### 2.5. Statistical Analysis

All eligible patients in the supplied files within the study window were included; cohort size was fixed by disease frequency rather than by design, and no target sample size was set. The registry collects data prospectively at listing, at transplantation and at each mandated follow-up interval across all United States transplant centers, so ascertainment is prospective and multicenter although the analysis is retrospective. The 51 recipients represent, to the limits of registry coding, the complete national experience of liver transplantation for FLC over 23 years. Precision was characterized by confidence intervals and internal-validation summaries rather than by hypothesis tests. Continuous variables are reported as medians and interquartile ranges (IQRs), and categorical variables with observed denominators. Aalen–Johansen cumulative incidence accounted for competing waiting-list outcomes and death before reported recurrence [24]. Patient-level bootstrap confidence intervals used 2000 resamples for waiting-list outcomes and 5000 for recurrence. Kaplan–Meier survival estimates used Greenwood log–log intervals; reverse Kaplan–Meier methods estimated potential follow-up. Main survival curves stop at five years and recurrence curves at three years, retaining at least one-third of the original cohort at risk. Descriptive summaries did not impute missing values; model imputation was training-sample specific. Analyses used Python 3.14 and R 4.5.1, with independent checks of cohorts, endpoints and calculations. Molecular evidence informed the discussion only and was not linked to registry patients.

## 3. Results

### 3.1. Study Population and Diagnostic Support

**Figure 1:**
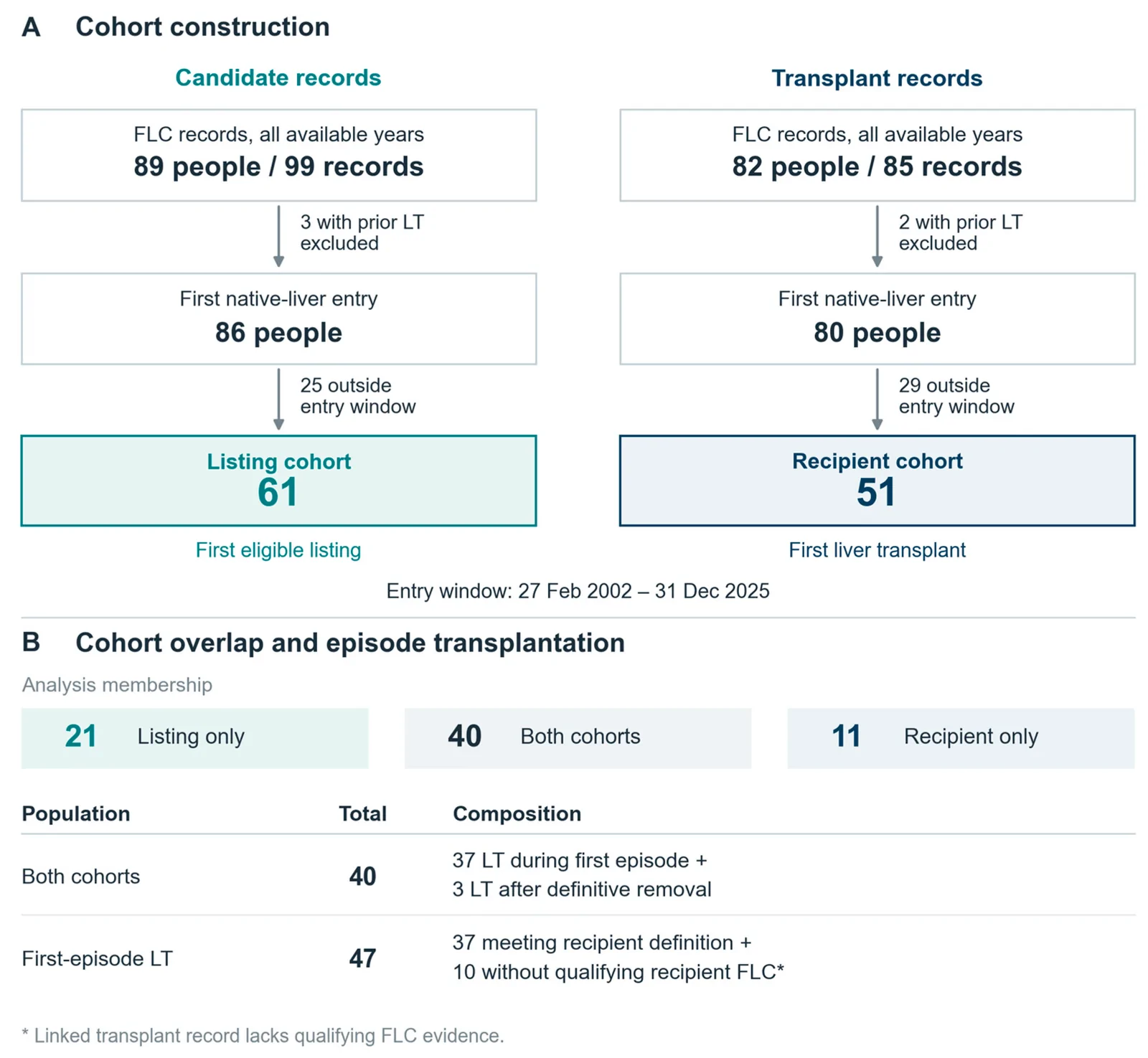
Cohort construction, analysis membership and first-episode transplantation. (**A**) FLC was identified independently in candidate and recipient diagnosis records. The earliest qualifying record was selected across available years before excluding previous liver transplantation and applying the entry window. The primary cohorts contained 61 listed candidates and 51 first-transplant recipients. (**B**) Membership refers to inclusion in the analyses. Of 40 patients in both cohorts, 37 underwent transplantation during the first listing episode and 3 after a definitive first-episode removal. Of 47 first-episode transplants, 37 met the recipient definition and 10 lacked qualifying FLC evidence in transplant records. Earlier definitive removal remained the waiting-list endpoint even when transplantation occurred later. FLC, fibrolamellar carcinoma; LT, liver transplantation.

The study included 61 listed candidates and 51 first-transplant recipients, with 40 patients represented in both cohorts (Figure 1). Of 47 candidates transplanted during their first listing episode, 37 met the recipient definition and 10 lacked qualifying FLC evidence in their transplant records. Three patients in both cohorts underwent transplantation after definitive first-episode removal.

Median age was 21.8 years at listing and 20.8 years at transplantation; approximately one-third of each cohort was younger than 18 years. Median laboratory MELD was 8 at listing and 9 at the last available candidate assessment among recipients. There were 38 deceased-donor and 13 living-donor transplants; 49 involved the liver alone and 2 included additional organs (Table 1).

Values are n (%) or median (interquartile range). The cohorts overlap in 40 people; they are not independent comparison groups. Chronic liver disease codes are administrative co-diagnoses and do not confirm cirrhosis; an absent code does not exclude liver disease. Laboratory scores use the initial candidate value for the listing cohort and the last available candidate value for recipients; the latter is not necessarily a transplant-day measurement. MELD and PELD are shown separately: MELD, model for end-stage liver disease; PELD, pediatric end-stage liver disease. No between-cohort significance tests were performed.

FLC was the center-reported pathology-confirmed discharge diagnosis in 42 of 51 recipients (82%). Either recipient diagnosis field contained code 4402 in 48 recipients, and 3 qualified through explicit FLC text. Nine candidates had a prelisting biopsy-confirmation date in an HCC exception field without a separate subtype result. Structured tumour assessments were available in 14 candidates. Median alpha-fetoprotein was 6.0 ng/mL, extrahepatic spread was recorded as ruled out in 14/14 and macrovascular involvement in 13/14. Original tumour measurements were available in 9, with median maximum diameter 8.3 cm on imaging performed a median of 227 days before listing (Tables S2, S8 and S9).

### 3.2. Access to Transplantation

During the first listing episode, 47 candidates underwent transplantation, 6 died or were removed for deterioration, 7 had another removal and 1 was censored. One-year transplantation incidence was 76.5% (95% CI 65.9–86.6), death or deterioration was 8.3% (95% CI 1.7–15.6), and other removal was 10.1% (95% CI 3.3–18.0). Median waiting time among the 47 transplanted during that episode was 22 days (IQR 12–102). Three candidates remained at risk for a first-episode exit at one year (Figure S1).

Exception applications were documented for 34 candidates, with dated effective priority periods for 31. The first recorded application occurred a median of 1 day after listing and the first effective period after 10 days. Effective exception periods covered 2384 of 6514 observed waiting-list patient-days, so that roughly a third of observed waiting time was spent with active exception priority (Tables S6 and S7).

### 3.3. Survival and Reported Recurrence

**Figure 2:**
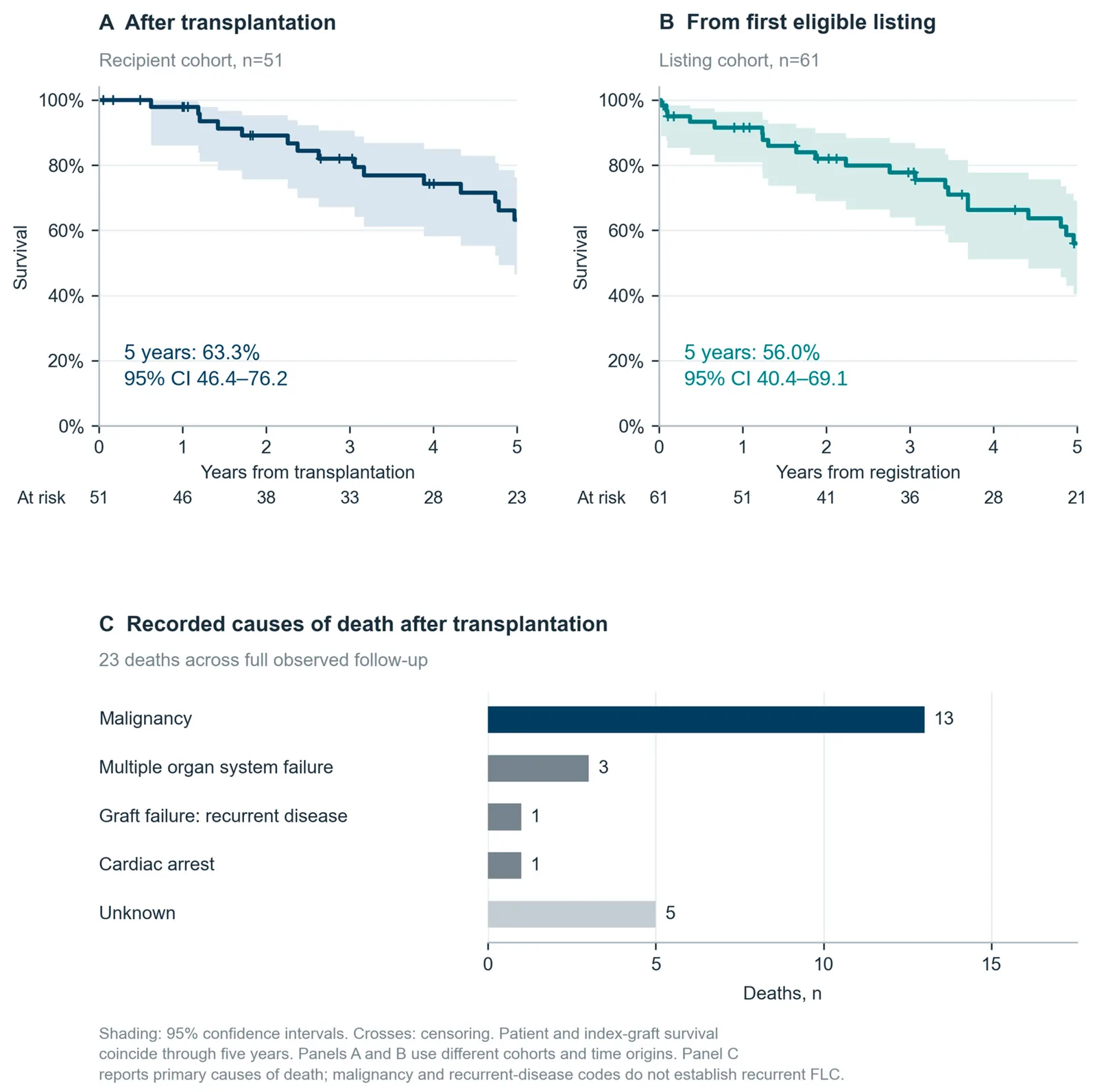
Survival and recorded causes of death. (**A**) Patient survival after the first liver transplant; all-cause index-graft survival coincides through five years. (**B**) Overall survival from listing, continuing through transplantation and later records. Shading indicates 95% Greenwood log–log confidence intervals, crosses indicate censoring, and numbers below the curves indicate patients at risk. Panels A and B use separate cohorts and time origins. (**C**) Recorded primary causes among 23 recipient deaths across complete follow-up, not only deaths within five years. Malignancy codes do not distinguish recurrent FLC from other malignancies. Recurrent-disease graft failure is a separate category without a specified tumour diagnosis.

After a median potential follow-up of 8.1 years, 23 recipients had died. Survival after transplantation was 97.9%, 82.0%, and 63.3% (95% CI 46.4–76.2) at one, three, and five years, respectively; 23 recipients remained at risk at five years (Figure 2A). Survival from listing was 91.6%, 77.7%, and 56.0% (95% CI 40.4–69.1), respectively, with median potential follow-up of 8.2 years and 21 candidates at risk at five years (Figure 2B). Malignancy was the recorded primary cause in 13 of 23 recipient deaths (Figure 2C).

Patient and all-cause graft survival coincided through five years. Across complete follow-up, 24 index grafts were lost: 20 deaths without preceding recorded graft failure, 3 deaths with same-day graft failure, and 1 retransplantation without observed death. No deaths were recorded within 90 days; status at that horizon was ascertainable for 49 recipients, while 2 were censored earlier. Other recorded causes of death were multiple-organ-system failure in three patients, graft failure attributed to recurrent disease in one, and cardiac arrest in one; cause was recorded as unknown in five (Table S3).

Five-year recipient survival was 64.4% (95% CI 45.7–78.1) among the 42 recipients with FLC in the pathological discharge field. Corresponding estimates were 64.7% when the dedicated FLC code was required, 64.2% when conflicting malignant codes were retained and 63.9% among isolated-liver recipients. Historical-entry analyses, death-date sensitivities, and calendar composition are reported in Tables S1, S4, S5, and S10.

**Figure 3:**
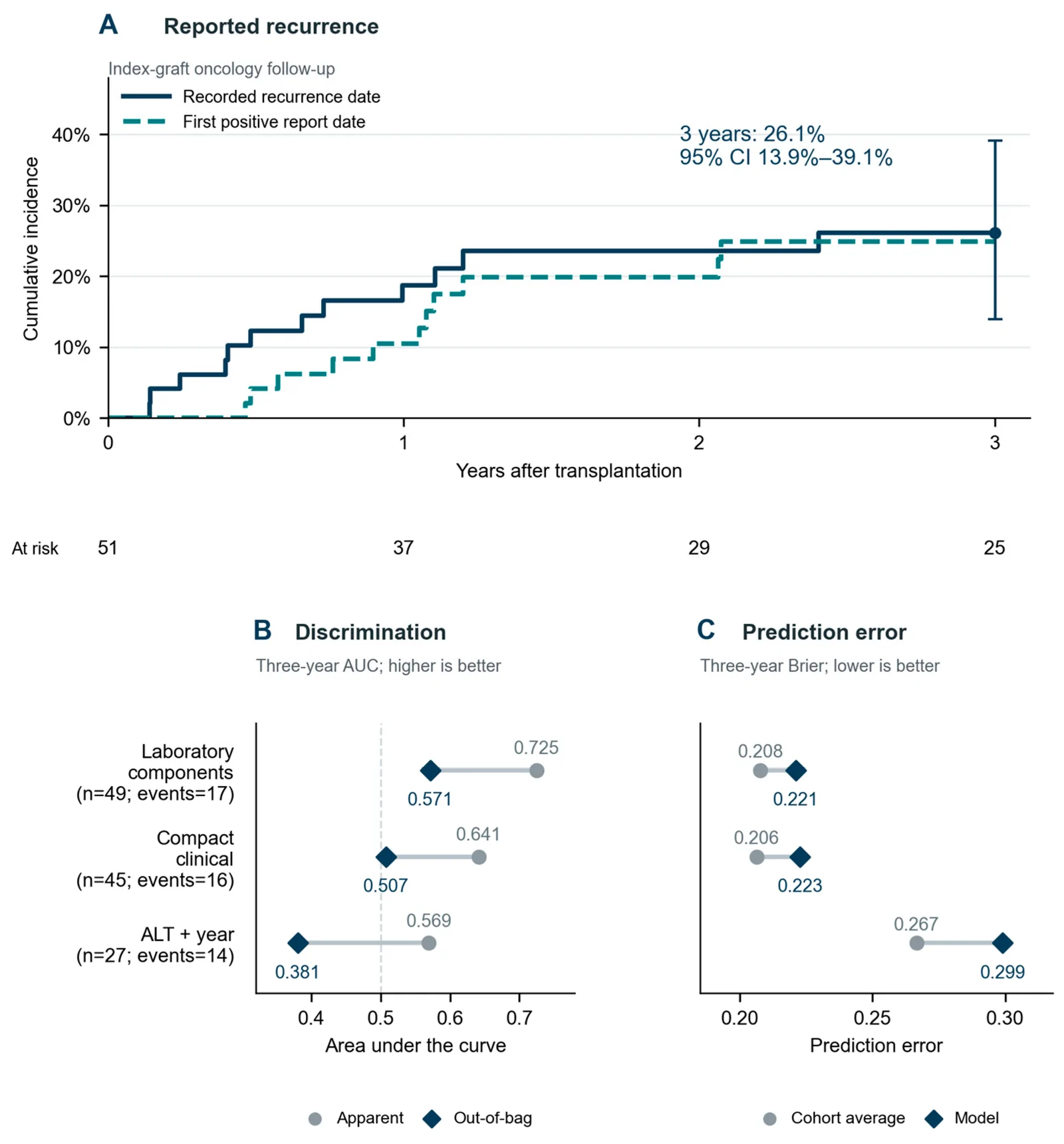
Reported recurrence and clinical prediction after transplantation. (**A**) Aalen–Johansen cumulative incidence using the recorded recurrence date and, in sensitivity analysis, the first positive report date; death without reported recurrence competes. The primary three-year interval is based on 5000 patient bootstrap samples. Two recipients without oncology follow-up are censored at day zero. (**B**) Apparent and mean out-of-bag three-year discrimination after 500 patient bootstrap samples. (**C**) Mean out-of-bag Brier score compared with the matched training-sample cohort-average Aalen–Johansen prediction. Lower Brier scores indicate less prediction error. All preprocessing and fitting used training patients only. The laboratory-component, compact clinical and ALT analyses contain 49/17, 45/16 and 27/14 recipients/recurrences, respectively; raw performance should not be compared across these different populations. Mean validation values are displayed without treating bootstrap-distribution quantiles as confidence intervals. None of these models establishes a transplant eligibility threshold.

Oncology follow-up was available for 49 of 51 recipients. Across available observation, 17 had reported recurrence of the pretransplant tumour, 7 died without reported recurrence, and 27 were censored, including the 2 without oncology follow-up. All seven competing deaths had an explicit negative oncology assessment on the recorded death date. Median potential oncology follow-up was 5.1 years. Twelve recurrences occurred within three years, giving a three-year cumulative incidence of 26.1% (95% CI 13.9–39.1), with 25 recipients remaining at risk (Figure 3A). Five recurrences were recorded later. Thirteen of the 17 reported recurrences were coded to the liver or biliary tract; restricting the endpoint to these gave a three-year cumulative incidence of 17.8% (95% CI 7.1–29.8) from 8 events, with other or unspecified sites contributing 8.3% (95% CI 2.0–16.7) as a competing event. Censoring at the last vital-status contact instead of the last negative oncology assessment gave a three-year cumulative incidence of 25.4% (95% CI 13.4–38.1). This and the other endpoint and ascertainment sensitivities are provided in Table S11.

### 3.4. Clinical Recurrence Models

Bilirubin, international normalized ratio, creatinine, and albumin were recorded in all 51 recipients, and sodium in 46. The latest pretransplant status began a median of 3 days before transplantation (IQR 1–16.5). The laboratory-component model included 49 recipients with 17 recurrences; the compact MELD-based model included 45 with 16 recurrences. Apparent three-year AUCs of 0.725 and 0.641 fell to mean bootstrap-validation AUCs of 0.571 and 0.507, respectively (Figure 3B). Prediction error exceeded the matched cohort-average prediction for both models; mean Brier scores were 0.221 versus 0.208 and 0.223 versus 0.206 (Figure 3C). In 25 of 49 recipients under the laboratory model and 22 of 45 under the compact model, the higher- or lower-risk assignment was not reproduced in at least 80% of the samples in which that recipient was omitted. These were the recipients who did not retain the same assignment in at least 80% of their omitted-sample evaluations (Figure S3; Tables S12–S17).

ALT data were available for 27 recipients transplanted during 2002–2014, including 14 with reported recurrence. The ALT-and-year model had a mean three-year validation AUC of 0.381 and a mean Brier score of 0.299, compared with 0.267 for the matched cohort-average prediction. The year-adjusted association per doubling of ALT was imprecise (hazard ratio 0.94, 95% CI 0.61–1.45) and changed after exclusion of a questionable source value (hazard ratio 1.54, 95% CI 0.81–2.92). Demographic benchmarks and sensitivity analyses likewise did not establish reproducible higher- and lower-risk groups, although the age-and-sex benchmark reached a mean three-year validation AUC of 0.538, above that of the compact clinical model (Figure S2; Tables S14–S17).

### 3.5. Explant Tumour Extent

The liver explant file yielded 44 eligible recipients. Ten had an index-transplant explant form and eight had complete core tumour fields. Four had recorded vascular invasion, nodal involvement or extrahepatic spread, and 4 had explicit absence of all three; 2 were unclassified. Recurrence was reported in 2 of the 4 recipients with an extent feature and none of the 4 with explicit absence over available follow-up (Table 2). Both recipients with recurrence had vascular invasion recorded as absent; one had nodal involvement and the other extrahepatic spread. At three years, the extent-positive group had one recurrence, two recipients with a negative assessment covering the horizon, and one with unknown status. The explicit-absence group had no reported recurrence, three patients with a negative assessment covering three years and one censored earlier. Its median negative oncology observation was 9.9 years (range 2.7–12.1). Capture, timing, and individual tumour features are detailed in Tables S18–S20.

Among 44 eligible recipients in the liver explant file, 10 had an index-transplant explant form and 8 had complete core tumour fields. Extent features were microvascular or macrovascular invasion, regional nodal involvement and extrahepatic spread. Missing fields were not classified as absent. The three three-year rows are mutually exclusive and sum to each group denominator; one extent-positive recipient with unknown status at three years had a later reported recurrence. Negative oncology observation ends at the last explicit negative assessment and is not extended by later vital contact or by negative forms after recurrence. Recurrence was registry reported, not centrally adjudicated FLC. These postoperative findings are exploratory and do not constitute pretransplant risk categories. The explant file was drawn from the same registry as the primary cohorts but carried a separately encrypted identifier; the two were not pooled. No between-group significance test was performed.

## 4. Discussion

We found that liver transplantation was followed by prolonged survival in selected patients with FLC. Five-year survival was 63.3%, with a similar estimate among recipients whose pathological discharge diagnosis confirmed FLC. Reported recurrence of the pretransplant tumour reached 26.1% at three years, and malignancy was the leading recorded cause of death. Routine clinical and laboratory models did not provide reliable recurrence discrimination after internal validation, a result that the small number of events leaves inconclusive. We treat each finding as an exploratory estimate from a small national cohort rather than a confirmed effect.

This study adds outcomes after acceptance for listing and recurrence reconstructed from linked oncology forms to the national recipient experience reported by Atienza and colleagues [14]. Their one-, three-, and five-year survival estimates were 96%, 80%, and 48%, against 97.9%, 82.0%, and 63.3% here. The difference at five years cannot establish improvement. The two series overlap in time (1988–2013 and 2002–2025) and draw on the same national records, but cohort definition and follow-up may differ. We required qualifying FLC evidence in the transplant record, pathology-supported in 82% of recipients, and reconciled death dates across registry, center, and Social Security sources. Their five-year estimate also lies within our 95% confidence interval. Both studies show, however, that prolonged survival is achievable, and the earlier surgical experience of Pinna and colleagues included long survivors after transplantation despite recurrence [11]. Recurrence is also common after resection [29], and paediatric data caution against treating FLC as indolent [30]. Recurrence alone therefore does not establish that the operation conferred no benefit.

Among candidates transplanted during the first episode, median waiting time was 22 days; across the listing cohort, one-year transplantation incidence was 76.5%. The latter includes candidates whose episode ended without transplantation and is more useful for counseling at listing. Death or removal for deterioration occurred in 8.3% by one year despite generally low liver-disease scores. After transplantation, five reported recurrences occurred beyond three years, supporting continued oncologic surveillance.

The prediction analyses should be read with particular caution. Neither clinical model reduced prediction error relative to its matched cohort-average estimate after internal validation, and historical ALT measurements did not show a stable association. These models were developed on 14 to 17 events, an order of magnitude below current sample-size recommendations [28], and without the tumour variables most likely to carry prognostic information. They cannot separate an uninformative predictor set from an informative one estimated too imprecisely, and we do not consider them clinically applicable. Disease distribution and treatment response are the more plausible candidates; outcomes have been related to the number of intrahepatic tumours, metastatic status, and treatment modality [13,31], and the current guidelines frame transplantation around unresectable, liver-confined disease [17]. The tumour-burden thresholds for conventional HCC were themselves derived in 48 and 70 transplanted patients, cohorts of the same order as the largest FLC transplant series [32,33].

The explant analysis is similarly limited. Both reported recurrences occurred in recipients with nodal involvement or extrahepatic spread, while none was reported among the four with all three extent features explicitly absent. With four recipients per group, these counts only indicate the variables a prospective study should capture and cannot define a pretransplant cutoff. Diagnostic review is equally important; in a Dutch nationwide review, expert reassessment revised the diagnosis in 38 of 54 historical cases [34], and the same concern applies to any registry-coded diagnosis, even in a cohort as young as this one.

External molecular studies identify candidates for such work (Table S21). The recurrent DNAJB1::PRKACA fusion supports diagnosis in the appropriate morphological setting [4,5,6] and is sufficient to generate tumours resembling FLC in mouse liver [35,36], but it has not been validated to distinguish post-transplant recurrence risk. An eight-gene expression signature [37], LINE-1 hypomethylation [38], and tumour-informed circulating tumour DNA [39] have been associated with outcome in resected or advanced disease, but none has been validated in transplant recipients, and current reviews continue to allocate transplantation on disease extent alone [40].

This study has important limitations. First, the analysis was based solely on registry data. The de-identified registry release does not permit linkage to the medical records of the treating centers, and no source data were obtained from them. Structured tumour staging reached the registry only through the exception application (14 of 61 candidates) and the explant pathology form (10 of 44 recipients in the explant analysis), so that tumour size and number, vascular and nodal status, extrahepatic disease, previous resection, locoregional or systemic therapy and response, and serial alpha-fetoprotein were unknown for most of the cohort. Second, and as a direct consequence, the predictive analyses could use only routinely recorded laboratory and clinical variables. In the absence of these tumour data, their value is limited, and they should not inform patient selection. Third, 61 candidates, 51 recipients and 17 reported recurrences produce wide confidence intervals around every estimate. Fourth, center-reported pathological diagnoses could not be centrally reviewed, recurrence depended on explicit oncology reporting, five causes of death were unknown, and living-donor and deceased-donor recipients, who numbered 13 and 38, could not be analysed separately. The explant file, drawn from the same registry, does not provide independent validation. Finally, patients never referred or listed, and comparable patients managed without transplantation, were not represented; these cohorts cannot estimate causal transplant benefit. Because FLC accounts for under two per cent of hepatocellular carcinoma in the United States [2,3], a cohort of this size is close to what the national registry can yield; the questions it leaves open will require a multicenter study with access to center-level clinical, imaging, and pathology records.

## 5. Conclusions

In this exploratory national registry analysis, most candidates accepted for listing with FLC reached transplantation, with a one-year incidence of transplantation of 76.5% and a median waiting time of 22 days, and survival at five years after transplantation was 63.3%. We report these as uncontrolled single-arm observations, and they cannot establish that transplantation conferred benefit. They describe what followed a decision to transplant in current national practice, in a cohort defined by registry diagnosis rather than by adjudicated resectability. Recurrence was common, however, and sometimes late; so, oncologic surveillance should continue well beyond the first three post-transplant years. The available routine pretransplant measurements, recorded without tumour staging for most recipients, did not establish reliable recurrence-based selection groups. With 51 recipients and 17 reported recurrences, neither can this analysis establish that such variables are uninformative. The prediction results are hypothesis-generating, and no threshold derived here should be used to offer or withhold transplantation. We therefore conclude that selection should remain multidisciplinary and oncologically focused. A multicenter transplant cohort with expert diagnostic review, dated staging and treatment response, tissue and serial blood sampling, and adjudicated recurrence is needed to test the added value of biological markers. Even a reproducible prognostic marker would require comparison with outcomes under alternative treatments before it could justify excluding a patient from transplantation.

## Figures and Tables

**Table 1:** Clinical and transplant characteristics of patients with FLC.

Characteristic	Listing Cohort	Recipient Cohort
Patients	61	51
Age, years	21.8 (16.9–35.2)	20.8 (16.0–33.2)
Age < 18 years	20 (33)	18 (35)
Female sex	32 (52)	25 (49)
Co-recorded chronic liver disease code	3 (5)	2 (4)
Laboratory MELD: patients with this score type	55	47
Laboratory MELD score	8.0 (7.0–12.0)	9.0 (7.0–15.0)
Laboratory PELD: patients with this score type	6	4
Laboratory PELD score	−5.5 (−7.8 to −4.0)	−1.0 (−4.5 to 3.8)
Donor age, years	Not applicable	28.0 (18.0–43.0)
Deceased donor	Not applicable	38 (75)
Living donor	Not applicable	13 (25)
Isolated liver transplant	Not applicable	49 (96)
Transplant involving additional organs	Not applicable	2 (4)
FLC as pathology-confirmed discharge diagnosis	Not applicable	42 (82)

**Table 2:** Recorded explant extent and recurrence in the SRTR liver explant file.

Characteristic	Any Extent Feature Present	All Three Explicitly Absent	Incomplete Classification
Recipients with an explant form	4	4	2
Reported recurrence over available follow-up, n	2	0	0
Recurrence within three years, n	1	0	0
Explicit negative observation covering three years, n	2	3	1
Oncology status unknown at three years, n	1	1	1
Competing death before recurrence, n	0	0	0
Negative oncology observation among recipients without reported recurrence, median (range), years	10.4 (8.2–12.7)	9.9 (2.7–12.1)	2.5 (1.0–4.0)

## Data Availability

Restrictions apply to the availability of these data. The data were obtained from the Scientific Registry of Transplant Recipients (SRTR) and are available from SRTR with the permission of SRTR, under the terms of a data use agreement; individual-level records cannot be redistributed by the authors. The aggregate results reported in this manuscript and its Supplementary Materials contain no individual-level data. Analysis code is available from the corresponding author on reasonable request.

## Supplementary Materials

The following supporting information can be downloaded at: https://www.mdpi.com/article/10.3390/cancers18203335/s1, Tables S1–S21 and Figures S1–S3, containing detailed cohort definitions, endpoint and death-date sensitivity analyses, exception-priority detail, full model specifications and coefficients, internal-validation summaries, explant capture, individual tumour features, and summary of external molecular evidence.
