# Supplementary material for: Liver Transplantation for Fibrolamellar Carcinoma: National Registry Analysis and Reported Outcomes
Source: Cancers (Basel). 2026 Oct 15;18(20):3335. doi: 10.3390/cancers18203335 (PMC13702327; doi:10.3390/cancers18203335)
Supplement: Supplementary file 1 [file cancers-18-03335-s001.zip › cancers-4610801-supplementary.pdf]

Data source and entry definitions

The source release was SRTR pubsaf2606. Candidate, recipient, follow-up, exception and status-history records were linked through the supplied person and registration identifiers. Primary entry was restricted to February 27, 2002–December 31, 2025; observed follow-up was capped at December 31, 2025. The entry window was applied after selecting the earliest eligible record across all available history. An earlier qualifying record excluded a later re-entry from the main incident cohort.

Candidate records required liver waiting-list organ coding, an observed listing date and no prior-liver-transplant flag. CAN_SOURCE=L records represented never-waitlisted living-donor recipients and were excluded from listing analyses. Recipient eligibility required no previous-liver-transplant flag and a selected REC_TX_DT equal to the earliest observed liver transplant date. All ages were eligible. Liver-containing multiorgan procedures were included initially and excluded in an isolated-liver sensitivity analysis.

Diagnosis algorithm

CAN_DGN/CAN_DGN2 supplied candidate primary/secondary diagnosis codes. REC_DGN supplied the transplant primary diagnosis; REC_DGN2 supplied the pathology-confirmed liver diagnosis at hospital discharge. Code 4402 identified FLC. Generic HCC/hepatoma codes 4400/4401 were compatible with a recorded subtype. Codes 4403, 4404, 4405, 4420 and 4430 specified competing tumor categories and excluded a record from the primary phenotype when co-recorded with FLC evidence. Such records could enter the expanded sensitivity. Other-primary-malignancy code 4410 required explicit qualifying text when used to establish FLC.

The reviewed 50-character diagnosis-other fields accepted explicit FLC/carcinoma wording and documented spelling variants, including isolated fibrolamellar wording in a diagnosis field. A phrase naming fibrolamellar sarcoma without carcinoma evidence was excluded. Candidate and recipient eligibility were determined separately. No diagnosis became listing-eligible because FLC appeared later in a recipient record. These are algorithmic record definitions, not central pathology adjudication; subtype recognition at the original entry date cannot be verified.

In the dedicated-code analysis, the earliest code-supported record was selected anew before date restrictions. It could therefore differ from the primary text-or-code entry and need not form a nested subset. The expanded definition relaxed conflicting-tumor exclusions; the all-era definition removed the lower calendar bound while retaining source-specific phenotype and first-native requirements. The additional pathology-restricted analysis required REC_DGN2=4402 on the index transplant. It uses a center-reported pathological diagnosis; slides and molecular results were not rereviewed by the investigators. Survival across cohort definitions is shown in Table S1.

Table S1. Survival across all cohort definitions

Definition	Time origin	n	1 year, % (95% CI)	3 years, % (95% CI)	5 years, % (95% CI)
Primary definition	Listing	61	91.6 (80.9-96.4)	77.7 (64.0-86.8)	56.0 (40.4-69.1)
Primary definition	Post-transplant	51	97.9 (86.1-99.7)	82.0 (67.1-90.6)	63.3 (46.4-76.2)

Definition	Time origin	n	1 year, % (95% CI)	3 years, % (95% CI)	5 years, % (95% CI)
Pathology-confirmed discharge FLC	Post-transplant	42	100.0 (CI not estimable)	83.3 (66.5-92.1)	64.4 (45.7-78.1)
FLC code required	Listing	53	90.4 (78.5-95.9)	75.1 (60.2-85.1)	56.3 (39.9-69.9)
FLC code required	Post-transplant	48	97.8 (85.6-99.7)	81.1 (65.7-90.1)	64.7 (47.5-77.5)
Conflicting malignancy retained	Listing	61	91.6 (80.9-96.4)	77.7 (64.0-86.8)	56.0 (40.4-69.1)
Conflicting malignancy retained	Post-transplant	52	98.0 (86.4-99.7)	82.4 (67.8-90.8)	64.2 (47.5-76.8)
Isolated liver transplant	Post-transplant	49	97.8 (85.6-99.7)	83.6 (68.6-91.8)	63.9 (46.5-77.0)
All available eras	Listing	86	90.4 (81.6-95.1)	74.2 (62.9-82.6)	57.2 (44.7-67.8)
All available eras	Post-transplant	80	94.9 (86.9-98.0)	81.1 (70.2-88.4)	59.9 (47.3-70.4)

Each row uses its own eligible cohort and time origin. All-era cohorts include the historical experience. The pathology-restricted one-year estimate is 100%, with incomplete follow-up at that horizon; a nondegenerate confidence interval is not estimable by the specified method. The subgroup is not an independent validation sample.

Pathological diagnosis and additional clinical outcomes

For liver recipients, REC_DGN2 is the center-reported pathology-confirmed liver diagnosis at hospital discharge. The release metadata and the official Adult Liver Transplant Recipient Registration worksheet corroborate this organ-specific meaning (https://unos.org/wp-content/uploads/Adult-TRR-Liver.pdf, accessed September 3, 2026). Code 4402 specifies fibrolamellar carcinoma. This field provides a pathological diagnosis but not slide images, detailed explant staging or molecular assay results. The separate REC_HISTO dataset contains histocompatibility and crossmatch information, rather than tumor histology. Diagnostic support is summarized in Table S2.

Table S2. Pathological diagnostic support in the national cohorts

Measure	Patients or records
Recipients with FLC in the pathology-confirmed discharge field	42 (82)
Recipients with FLC as the transplant primary diagnosis	42 (82)
Recipients with FLC in both diagnosis fields	36 (71)
Recipients with FLC in either diagnosis field	48 (94)
Other discharge pathology code: 999 (999: OTHER SPECIFY)	4

Measure	Patients or records
Other discharge pathology code: 4400 (4400: LI:PLM: HEPATOMA - HEPATOCELLULAR CARCINOMA)	4
Recipients missing a discharge pathology code	1
Listed candidates with a biopsy-confirmation date	9 (15)
Biopsy dates preceding listing	9
Biopsy dates during the first listing episode	0
Biopsy dates after the first listing episode	0

REC_DGN2 records the center-reported pathology-confirmed liver diagnosis at hospital discharge. Code 4402 identifies fibrolamellar carcinoma. Primary and pathology diagnosis counts overlap. This structured pathological diagnosis is distinct from central slide review or molecular testing. CANHX_MPXCPT_HCC_BIOPSY_DT records a biopsy confirming HCC; the date field does not itself specify the fibrolamellar subtype. Nine forms in nine candidates contained biopsy dates. Missing dates do not establish that a biopsy was not performed.

The pathology-restricted analysis selected existing primary recipients whose index-transplant REC_DGN2 equaled 4402. No patient was reclassified at listing. The time origin, clinical-first death hierarchy, follow-up and endpoint definitions were unchanged. Its Kaplan–Meier estimates and log-log confidence limits were checked using SciPy's independent empirical-survival implementation. The one-year estimate reached 100% with incomplete fixed-horizon follow-up, so a nondegenerate confidence interval was not estimable by the specified method.

Biopsy confirmation was examined using CANHX_MPXCPT_HCC_BIOPSY_DT on exception forms already linked to the first continuous listing episode. The field records a date of biopsy confirming HCC without encoding its subtype. All nine populated dates preceded listing; none followed the episode. These dates therefore document prior diagnostic work, rather than biopsies newly performed while waiting.

Recorded primary cause of death was taken from PERS_OPTN_COD only when PERS_OPTN_DEATH_DT equaled the validated patient death date. The selected code was checked against TFL_COD on a same-date deceased-status follow-up. All 23 deaths had one unambiguous date-matched code and agreement between these sources. Codes 4850, 4851 and 4856 were grouped as malignancy; 4604, graft failure from recurrent disease, remained separate because it does not name a tumor. Multiple-organ-system failure, cardiac arrest and unknown causes retained their source categories. The analysis describes the recorded primary cause rather than contributing causes or adjudicated recurrent FLC.

Early deaths were counted through 30 and 90 days from transplantation. Horizon status was known if a patient had an observed death or follow-up reaching that horizon. Patients censored earlier remained incompletely ascertained, so the absence of recorded early deaths was not reported as a complete-cohort zero mortality rate. Recorded causes of death and early outcome ascertainment are shown in Table S3.

Table S3. Recorded causes of death and early outcome ascertainment

Registry cause code	Cause or measure	Deaths, n / value
998	998: UNKNOWN	5

Registry cause code	Cause or measure	Deaths, n / value
4604	4604: LI:GRAFT FAILURE:RECURRENT DISEASE (NON-HEPATITIS)	1
4626	4626: LI:CARDIAC ARREST	1
4660	4660: LI:MULTIPLE ORGAN SYSTEM FAILURE	3
4850	4850: LI:MALIGNANCY: PRIMARY OTHER SPECIFY	3
4851	4851: LI:MALIGNANCY: METASTATIC OTHER SPECIFY	9
4856	4856: LI:MALIGNANCY	1
—	All deaths	23
—	Malignancy codes combined	13
—	Recorded deaths within 30 days	0
—	Status known at 30 days	50/51
—	Censored before 30 days	1
—	Recorded deaths within 90 days	0
—	Status known at 90 days	49/51
—	Censored before 90 days	2

Cause counts describe all 23 observed recipient deaths over complete follow-up, not five-year deaths. PERS_OPTN_COD was selected only when its death date matched the validated death date; every code agreed with the same-date deceased-status TFL_COD. Malignancy comprises codes 4850, 4851 and 4856. Code 4604 denotes graft failure from recurrent disease without specifying a tumor and is not combined with malignancy. Unknown codes remain a separate category. Absence of a recorded early death does not imply complete 30- or 90-day ascertainment.

An initial feasibility screen preceded the final diagnosis lock, and aggregate outcomes had been observed during that screen. Source-specific phenotype adjudication was then performed without individual outcome dates or status. This was not a prospectively registered study. Calendar, oncology-capture and pathology/clinical-outcome summaries were added transparently during review and did not change the primary cohort or endpoint definitions.

Continuous listing episode and competing outcomes

The episode began at the selected FLC-eligible listing. Concurrent or overlapping native-liver registrations were united. A terminal administrative transfer, CAN_REM_CD=7, linked to a subsequent episode only with documented same-day or next-day continuation; an unlinked transfer was censored. A definitive removal ended the episode, and later relisting did not erase the first outcome. This endpoint addresses the initial continuous episode, not whether a person ever received a transplant over their lifetime.

Verified linked liver transplantation was checked against removal timing and took precedence in same-day ties. CAN_REM_CD values 8, 13, 21 and 23 identified death or clinical deterioration. Other definitive removal codes were grouped as other removal. A transplant-removal code without a linked transplant date was treated as an error requiring reconciliation. Death after other removal was not automatically reassigned to the competing death/deterioration endpoint. Open registrations required CAN_ENDWLFU supporting the administrative horizon. See Table S4 for the primary survival and competing-outcome estimates.

Table S4. Primary survival and competing-outcome estimates

Endpoint	Year	Estimate, % (95% CI)	At risk, n	Total events
Survival from listing	1	91.6 (80.9-96.4)	51	30
Survival from listing	3	77.7 (64.0-86.8)	36	30
Survival from listing	5	56.0 (40.4-69.1)	21	30
Post-transplant patient survival	1	97.9 (86.1-99.7)	46	23
Post-transplant patient survival	3	82.0 (67.1-90.6)	33	23
Post-transplant patient survival	5	63.3 (46.4-76.2)	23	23
Post-transplant index-graft survival	1	97.9 (86.1-99.7)	46	24
Post-transplant index-graft survival	3	82.0 (67.1-90.6)	33	24
Post-transplant index-graft survival	5	63.3 (46.4-76.2)	23	24
Transplant incidence	1	76.5 (65.9-86.6)	3	Not applicable
Death/deterioration incidence	1	8.3 (1.7-15.6)	3	Not applicable
Other-removal incidence	1	10.1 (3.3-18.0)	3	Not applicable
Transplant incidence	3	76.5 (65.9-86.6)	1	Not applicable
Death/deterioration incidence	3	10.0 (3.3-18.0)	1	Not applicable
Other-removal incidence	3	11.8 (4.9-20.2)	1	Not applicable
Transplant incidence	5	78.2 (68.1-88.2)	0	Not applicable
Death/deterioration incidence	5	10.0 (3.3-18.0)	0	Not applicable
Other-removal incidence	5	11.8 (4.9-20.2)	0	Not applicable

Total events cover all observed follow-up. Survival CIs use Greenwood log-log calculations; cumulative-incidence CIs use 2,000 patient bootstrap samples. Waitlist risk sets comprise candidates without a first-episode endpoint. Five-year incidence carries the last Aalen-Johansen probabilities forward after the observed risk set is exhausted, assuming non-informative censoring; it does not provide a five-year waiting-list denominator. One-year incidence is the principal access estimate.

Figure S1 displays the first-year listing trajectory, its remaining risk set and the timing of captured exception applications. Exception timing is conditional on the corresponding dated record.

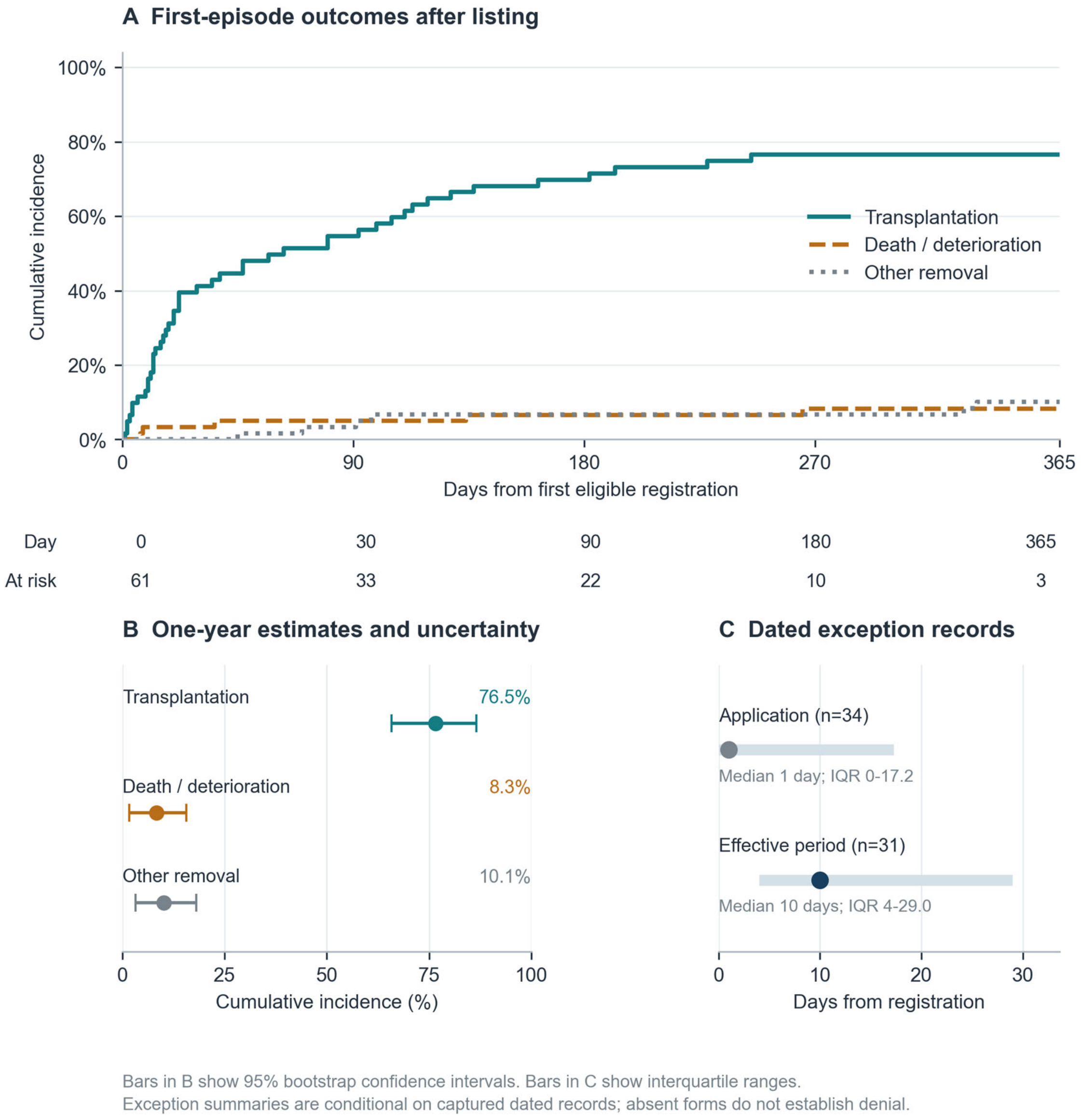

Figure S1. The first year after listing for FLC. (A) Cumulative incidence of transplantation, death or removal for clinical deterioration, and other removal, estimated with the Aalen–Johansen method. The separate risk strip shows candidates remaining without a first-episode exit. (B) One-year estimates with 95% patient-bootstrap confidence intervals. (C) Time from listing to the first documented exception application or effective priority period; points indicate medians and bars indicate interquartile ranges. Exception

summaries use patients with the respective dated records. Absence of a form does not establish denial or absence of an application.

Patient survival and death-date reconciliation

Person death ascertainment used PERS_OPTN_DEATH_DT, CAN_DEATH_DT, TFL_DEATH_DT and dated deceased-status records from TFL_PX_STAT/TFL_PX_STAT_DT. PERS_SSA_DEATH_DT was evaluated separately. Clinical dates had to agree; multiple clinical dates or multiple Social Security dates were unresolved errors. The primary rule used the concordant clinical date when available and the Social Security date otherwise. Explicit removal-for-death dates could recover an otherwise undated death. A deceased status without a dated source was not accepted as routine censoring.

The clinical-first hierarchy was finalized after source-quality review revealed discrepant dates. The sensitivity analysis used the earliest available source date and compared fixed-horizon estimates. Clinical and Social Security dates differed for 3 patients in the primary cohorts and 8 patients across all eligible eras. Discrepancies were retained in the quality log rather than silently choosing the date that favored a particular estimate. The effects of death-date selection are shown in Table S5.

Table S5. Sensitivity to death-source date selection

Endpoint	Year	Primary, %	Earliest source, %	Difference, percentage points
Listing: patient survival	1	91.571	91.597	+0.026
Listing: patient survival	5	56.028	56.044	+0.016
Recipient: patient survival	1	97.917	97.917	+0.000
Recipient: patient survival	5	63.294	63.294	+0.000
Recipient: all cause index graft survival	1	97.917	97.917	+0.000
Recipient: all cause index graft survival	5	63.294	63.294	+0.000
Listing: transplant incidence	1	76.545	76.575	+0.030
Listing: death deterioration incidence	1	8.323	8.283	-0.040
Listing: other removal incidence	1	10.088	10.095	+0.007
Listing: transplant incidence	5	78.226	78.257	+0.031
Listing: death deterioration incidence	5	10.004	9.965	-0.039

Endpoint	Year	Primary, %	Earliest source, %	Difference, percentage points
Listing: other removal incidence	5	11.770	11.777	+0.008

Primary deaths use concordant registry and center dates when available and Social Security Administration dates otherwise. The sensitivity uses the earliest dated source. Differences are sensitivity minus primary estimate. Extra decimal places show the small numerical changes rather than implying additional clinical precision.

Observed dates supporting patient follow-up included listing and removal events, dated active/inactive status changes, transplant and discharge dates, TFL_LAFUDATE and dated alive/dead follow-up status. Death after the analytic cutoff established survival through the cutoff. Otherwise follow-up ended at the latest observed date, subject to an open waiting-list registration with substantiated coverage. Overall survival from listing could continue after removal, transplantation or relisting; recipient survival continued across grafts. TFL_ENDTXFU was not treated as an observed survival date.

Index-graft outcome

All-cause graft loss was the earliest of death, index-graft failure or subsequent liver transplantation. Potential failure sources were REC_FAIL_DT, TFL_GRAFT_DT, index-graft TFL_FAIL_DT, subsequent REC_TX_DT and PERS_RETX as a corroborating repeat-transplant source. Failed-graft status without a date required recovery from a linked dated record. A failure date coinciding with death was retained as a graft endpoint but was not counted as graft loss preceding death.

Censoring was specific to the index graft and used observed index-graft follow-up. Later patient survival alone did not prove continued original-graft function. The composite includes patient death and cannot be interpreted as death-censored graft survival or recurrence-free survival. Registry coverage and graft-loss components are summarized in Table S6.

Table S6. Registry ascertainment, follow-up coverage and graft-loss components

Measure	Count
Listing cohort: dedicated-code support at entry	52
Listing cohort: explicit text support at entry	9
Recipient cohort: dedicated-code support at entry	48
Recipient cohort: explicit text support at entry	3
Missing age: listing / recipient	0 / 0
Missing laboratory score: listing / recipient	0 / 0
Recipients with index-graft follow-up forms	49 / 51
Status history: covered patient-days	6308
Status history: exception flag present, patient-days	2397
Status history: exception flag absent, patient-days	3911
Status history: unclassified patient-days	206

Measure	Count
All concurrent registrations inactive, patient-days	2307
Graft loss: death first	20
Graft loss: death and failure on the same day	3
Graft loss: failure or retransplant first	1
Clinical/SSA death-date discordance: primary people	3
Clinical/SSA death-date discordance: all-era people	8

Source support can overlap when both code and text are present; counts should not be assumed mutually exclusive. Missing follow-up forms do not imply missing vital status when other dated sources establish survival or death. Status-history flags are separate from dated approval-form periods. The three graft-loss components are mutually exclusive and total all observed index-graft losses. SSA, Social Security Administration.

Exception capture and dated periods

Exception forms were limited to registrations belonging to the first episode and applications recorded by its endpoint. CANHX_MPXCPT_ORIG_APPL_DT identified the original application date. CANHX_MPXCPT_BEGIN_DT and CANHX_MPXCPT_END_DT defined effective periods only for statuses whose supplied labels documented approved history. Missing begin dates were not inferred from a last-change field. An open effective period could continue to the episode endpoint only for a status supporting an active approval. Captured applications and effective periods are summarized in Table S7.

Table S7. Captured exception applications and effective periods

Measure	Value
Candidates with captured exception forms	34 (56)
Candidates without captured exception forms	27 (44)
Candidates with dated applications	34
Candidates with dated effective exception periods	31
Listing to first captured application, days	1.0 (0.0–17.2)
Listing to first dated effective period, days	10.0 (4.0–29.0)
Active exception person-days	2384
Total first-episode person-days	6514
Approved forms without an effective date	0
Forms without an application date	0
Denied/not-approved form records	13

Values are n (%) or median (interquartile range), unless stated otherwise. Percentages use the primary listing cohort. Missing forms do not establish absence of a request, ineligibility, or denial. Form counts are not patient counts and may include repeat applications. Dated effective periods were clipped to the first continuous registration episode and overlapping intervals were united. Effective-date summaries are conditional on a captured dated period; they do not estimate approval probability or an exception effect.

Approved-history status codes were 5, 10, 14, 16, 18, 30, 32, 33, 34, 35, 36 and 37. Effective periods were clipped to the episode and overlapping periods merged to avoid duplicate patient-days. Denied/not-approved records were summarized separately; they were not used to erase earlier documented approved time. The first application date and first effective date were reported separately. Status-history coverage, unknown intervals and HCC/other exception flags were independently summarized. These administrative categories were not used to determine tumor histology.

An interval's status at the terminal event describes timing, not a baseline exposure group. No regression model, landmark comparison or causal exception-effect estimate was fitted. Lack of a captured form means lack of observed form evidence. It does not prove that an application was never made or that priority never changed.

Oncologic field capture

The capture audit linked exception forms to registrations in each validated first continuous listing episode and excluded applications after its endpoint. HCC-labeled fields included alpha-fetoprotein (AFP), tumor number, macrovascular and extrahepatic assessments, resection candidacy and treatment dates. Linked current and original tumor tables supplied size-field coverage. Counts describe nonmissing fields by form and by patient; they do not measure the prevalence of an oncologic feature. Assessment and treatment dates could precede listing or occur during the episode, so capture was not interpreted as baseline staging.

Clinical characterization used the earliest application within the first listing episode containing a nonmissing value, selected separately for each field. Original maximum tumor diameter was the largest reported lesion within that form, not the largest measurement across follow-up. Conflicting values on the earliest application date would have triggered a validation failure; no such conflict occurred. AFP, tumor count, vascular and extrahepatic assessments, and resection candidacy shared the same 14 patients and forms. Original diameter was available in nine of those patients and came from the same selected form in eight. Measurement dates were retained separately from form dates: 11 AFP tests preceded listing and three occurred during the episode; all nine original imaging dates preceded listing. These measurements therefore describe the first captured clinical observations rather than a common listing-day baseline (Table S8).

Table S8. Clinical characterization from captured tumor assessments

Characteristic	Observed, n	Median (IQR) or n
AFP, ng/mL	14	6.0 (2.2–10.8)
Tumors reported: one	14	12
Tumors reported: two	14	1
Tumors reported: zero	14	1
Macrovascular involvement ruled out	14	13
Macrovascular involvement not ruled out	14	1
Extrahepatic spread ruled out	14	14
Not a resection candidate (N)	14	10

Characteristic	Observed, n	Median (IQR) or n
Other resection-candidacy response (A)	14	4
Original maximum tumor diameter, cm	9	8.3 (4.0–8.7)
AFP test before listing	14	11
Original imaging before listing	9	9

Each field uses the earliest eligible exception application with a nonmissing value. The first five fields share 14 patients; original diameter is available in nine of them. Maximum diameter is taken within the selected form. Measurement dates may precede form submission: original imaging preceded listing by a median of 227 days. These are field-specific observations, not a common baseline for all 61 candidates. A negative rule-out response does not prove invasion. Resection response A is not decoded in the supplied catalog and is not combined with N. A reported tumor count of zero does not establish absence of FLC or complete treatment response. AFP, alpha-fetoprotein; IQR, interquartile range.

Malignancy records were linked through same-release follow-up keys for recipient patients, with index and subsequent grafts checked separately. In this cohort, all 40 linked records belonged to the index graft. Linked follow-up status dates were within the analytic horizon. The 33 populated recurrence-date records fell between index transplantation and the horizon, with none after a recorded death. A follow-up status date was not assumed to be the date a form was filed.

The pre-existing-tumor categories included liver/biliary tumors, incidental hepatic/biliary tumors and other cancers. They did not provide FLC histologic adjudication. Repeated records may refer to the same clinical event, and populated indicator fields may include defaults. Missing forms or dates do not establish absence of recurrence. The initial field-completion audit is retained in Table S9; the subsequent source-linked reported-recurrence endpoint and its sensitivities are defined below. This endpoint describes reported recurrence of a pretransplant tumor in recipients with FLC, rather than centrally adjudicated recurrent FLC.

Table S9. Capture of oncologic fields in the primary cohorts

Cohort	Data element	Patients with capture, n/N (%)
Listing	Any captured exception form	34/61 (56)
Listing	Alpha-fetoprotein value	14/61 (23)
Listing	Number of tumors	14/61 (23)
Listing	Assessment of macrovascular involvement	14/61 (23)
Listing	Assessment of extrahepatic spread	14/61 (23)
Listing	Resection candidacy	14/61 (23)
Listing	Chemoembolization date	5/61 (8)
Listing	Radiofrequency ablation date	1/61 (2)
Listing	Surgical resection date	1/61 (2)
Listing	Current tumor size	14/61 (23)
Listing	Originally reported tumor size	9/61 (15)

Cohort	Data element	Patients with capture, n/N (%)
Recipient	Any linked malignancy record	20/51 (39)
Recipient	Recorded post-transplant recurrence date	17/51 (33)

Listing fields come from exception forms attached to native-liver registrations intersecting the first listing episode; applications after that episode are excluded. Tumor sizes are linked through the same-release exception-form keys. Capture means at least one nonmissing record, not a baseline measurement or a positive clinical finding. Malignancy records were linked through recipient follow-up keys; all captured records linked to index grafts. The recurrence dates shown fall after the index transplant and on or before December 31, 2025. Tumor classifications in these records are broad liver/biliary or other-cancer categories, without established FLC-specific adjudication. These counts measure data availability and do not estimate FLC recurrence incidence. Absent records do not establish absence of disease or treatment.

Estimation, uncertainty and quality checks

The release stores CAN_INIT_SRTR_LAB_MELD and CAN_LAST_SRTR_LAB_MELD as numeric CANDSTAT encodings. Values were mapped through the supplied MELD/PELD labels, while special allocation-status codes were excluded from numeric score summaries. CAN_INIT_SRTR_LAB_MELD supplied the listing laboratory score and CAN_LAST_SRTR_LAB_MELD the last recorded laboratory score for recipients. The accompanying M/P indicators separated MELD from PELD; the fields did not establish tumor burden or molecular diagnosis.

Follow-up time was computed in days; fixed-year horizons used 365.25 days per year. Aalen–Johansen cumulative incidence treated transplantation, death/deterioration and other removal as competing events, with events processed before censoring at tied times. Patient-level percentile bootstrap confidence intervals used 2,000 replicates and seed 20260903. Kaplan–Meier survival used Greenwood log-log intervals. When every person's fixed-horizon event status was known, exact Clopper–Pearson intervals were used. Boundary estimates with incomplete follow-up did not receive falsely precise degenerate intervals.

Reverse Kaplan–Meier estimated potential follow-up by exchanging event and censoring indicators. Estimates beyond observed support were suppressed. Main Kaplan–Meier displays retained at least one-third of the initial cohort at risk. The first-year competing-event display is interpreted with its remaining waiting-list risk set and uncertainty; most candidates leave that risk set through an observed event. Later competing-incidence estimates, if tabulated, should not be treated as new evidence of stable long-term access.

The calendar groups describe recruitment and available follow-up; they were not prespecified policy comparisons. Observed follow-up is elapsed time from cohort entry to death or censoring, whereas reverse Kaplan–Meier estimates potential follow-up. No period-specific survival probabilities or era-effect model was fitted. Calendar composition and observed follow-up are shown in Table S10.

Table S10. Calendar composition and observed follow-up of the primary cohorts

Entry period	Cohort	Patients, n	Observed follow-up, years
2002-2014	Listing	34	5.0 (1.7–10.5)

Entry period	Cohort	Patients, n	Observed follow-up, years
2015-2018	Listing	6	3.8 (1.9–6.4)
2019-2025	Listing	21	2.2 (1.0–4.3)
2002-2014	Recipient	28	6.6 (3.0–11.7)
2015-2018	Recipient	6	5.9 (4.4–7.8)
2019-2025	Recipient	17	2.7 (1.0–4.0)

Follow-up is the observed interval from cohort entry to death or censoring, summarized as median (interquartile range). It is not the reverse Kaplan-Meier estimate of potential follow-up. Calendar groups were added descriptively during manuscript review to expose the composition of pooled estimates. They are not policy-effect comparisons, and no period-specific treatment-effect model was fitted.

Checks addressed unique patients, earliest-entry selection, source-specific diagnosis, first-native status, nonnegative durations, linked transplantation, death-date agreement, post-removal follow-up, graft failure and retransplantation. Aggregate estimates were recomputed independently before final manuscript production. The survival and access analyses were descriptive; no post hoc power calculation or adjusted treatment-benefit estimate was used. Exploratory recurrence prediction and pathology analyses are specified below. The accompanying tables retain source fields and missingness so administrative coverage is distinguishable from clinical absence.

Reported recurrence: linkage, dates and competing outcomes

The recurrence extension linked malignancy records to their actual parent index-graft follow-up by TRR_FOL_ID. An event required TFL_MALIG=Y, TFL_MALIG_RECUR_TUMOR=Y and a valid MAL_RECUR_DT after index transplantation and on or before the administrative cutoff and observed death. The earliest qualifying date was used; repeated records were collapsed to a first event. The endpoint is reported recurrence of a pretransplant tumor among recipients with FLC. Broad liver/biliary and other-tumor categories do not centrally adjudicate recurrent FLC.

Primary non-event follow-up ended at the last index-graft TFL_PX_STAT_DT with status A, D or R and either TFL_MALIG=N or TFL_MALIG=Y with TFL_MALIG_RECUR_TUMOR=N. Unknown/missing responses, Not Seen and Lost to Follow Up did not establish a new negative assessment. Two recipients without oncology follow-up were censored at day zero for incidence estimation and excluded from prediction models. The 49 recipients with informative observation contributed 350 dated index-graft forms; 274 forms met the explicit-negative definition.

The official follow-up instructions concern newly diagnosed malignancy since the preceding assessment; persistent malignancy need not be re-reported. A later negative response therefore did not reverse an established recurrence. Four recipients had five negative interval forms between a retrospective recurrence date and its first positive report. Median date-to-report lag was 132 days (IQR 43-252). Two source dates exceeded the parent status date by one day; strict exclusion of those rows altered one earliest event date by 93 days.

Seven deaths without an earlier reported recurrence competed with recurrence. Each had an explicit negative oncology response on the death-status date. Two nevertheless carried metastatic-malignancy death codes; these were deaths without reported recurrence, rather than verified cancer-free deaths. Death

codes alone were not converted into recurrence events. Dated recurrence would take precedence in a same-day recurrence/death tie.

Cumulative incidence used Aalen-Johansen estimation and 5,000 person-level percentile bootstrap resamples, seed 20260903. Three years was the primary recurrence horizon and one year secondary. Five-year estimates are descriptive because only 15 of 51 recipients remained at risk in the primary analysis. Table S11 reports incidence, event counts and risk sets across the fixed sensitivity definitions.

Table S11. Cumulative incidence of reported recurrence and source-definition sensitivities

Analysis	n	Events	1 year, % (95% CI)	3 years, % (95% CI)	5 years, % (95% CI)	At risk, 1/3/5 years
Primary: recorded recurrence date	51	17	18.7 (8.3-30.4)	26.1 (13.9-39.1)	32.2 (18.3-46.8)	37/25/15
Discharge pathology FLC	42	14	14.9 (4.9-27.0)	23.9 (10.8-39.0)	30.7 (15.9-46.6)	32/21/14
Hepatic/biliary first recurrence	51	13	10.4 (2.1-20.1)	17.8 (7.1-29.8)	23.9 (11.3-37.6)	37/25/15
Discharge FLC + hepatic/biliary first recurrence	42	11	7.5 (0.0-16.9)	16.5 (5.3-30.0)	23.2 (9.8-38.3)	32/21/14
Exclude date-discordant source rows	51	17	18.7 (8.3-30.4)	26.1 (14.0-39.2)	32.3 (18.3-46.8)	37/25/15
First positive report date	51	17	10.5 (2.2-19.9)	24.9 (12.9-38.3)	33.9 (19.4-49.1)	41/26/15
All-graft oncology follow-up	51	17	18.5 (8.2-30.2)	25.7 (13.7-38.7)	31.6 (18.0-46.0)	38/26/16
Generic vital censoring	51	17	18.4 (8.2-30.0)	25.4 (13.4-38.1)	31.1 (17.6-45.0)	38/26/19

Death without reported recurrence competes in every analysis. Other/unspecified first recurrence additionally competes in the hepatic/biliary analyses. Total events cover all follow-up, not only the displayed horizons. CIs are 5,000-person-bootstrap percentile intervals. Five-year values are descriptive; the primary risk set is 15/51. Two recipients were censored at zero in the primary analysis. The primary definition has 17 recurrences, seven competing deaths and 27 censored recipients; 9 and 12 recurrences occurred by one and three years. The endpoint is reported pretransplant-tumor recurrence, not histologically adjudicated recurrent FLC.

Sensitivities restricted REC_DGN2 to FLC; used hepatic/biliary first-recurrence categories 15/16 while treating other/unspecified first recurrence as an additional competing event; excluded source-date/parent-date discordant rows; substituted the first positive report date; allowed same-person subsequent-graft oncology follow-up; or used generic vital follow-up for censoring. Later hepatic recoding did not replace

an earlier other-tumor event. Later-graft and generic-vital censoring were sensitivities because they can extend observation across an interval without explicit oncology assessment.

Clinical predictor availability and model populations

Laboratory components were taken from the latest index-registration status record with CANHX_BEGIN_DT strictly before transplantation. Actual bilirubin, INR and creatinine values were used rather than their MELD-bounded counterparts. Bilirubin, INR, albumin and creatinine were present in all 51 recipients, sodium in 46. The status record preceded transplantation by a median of 3 days (IQR 1-16.5); 43 records were within 30 days and 50 within 90 days. This timestamp identifies the recorded status snapshot, not the blood draw. Fourteen patients had identical panels on successive terminal status records, including four spanning more than 30 days; identical values were not assumed to represent either repeated testing or carry-forward.

Pretransplant hospitalization combined REC_MED_COND=1 (intensive care) and 2 (hospitalized outside intensive care), compared with 3 (outpatient). Waiting time came from the associated registration and did not measure duration of tumor observation or treatment response. The compact model used the latest strictly pretransplant status-history CANHX_SRTR_LAB_MELD and its M/P type, separately from the descriptive CAN_LAST summary score. Only MELD-type recipients entered this model (47 eligible, 45 with informative follow-up); four PELD-type recipients were not pooled with MELD. The component model included all 49 informatively followed recipients. Table S12 reports coverage and temporal constraints.

Table S12. Pretransplant predictor capture in the SRTR recipient cohort

Predictor	Source field	Observed / eligible	Missing or outside type	Recurrences among observed	Units	Timing/interpretation
Bilirubin	CANHX_BILI	51/51	0	17	mg/dL	Latest pre-LT status snapshot; blood-draw date unavailable
INR	CANHX_INR	51/51	0	17	ratio	Latest pre-LT status snapshot; blood-draw date unavailable
Albumin	CANHX_ALBUMIN	51/51	0	17	g/dL	Latest pre-LT status snapshot; blood-draw date unavailable
Creatinine	CANHX_SERUM_CREAT	51/51	0	17	mg/dL	Latest pre-LT status snapshot; blood-draw date unavailable

Predictor	Source field	Observed / eligible	Missing or outside type	Recurrences among observed	Units	Timing/interpretation
Sodium	CANHX_SERUM_SODIUM	46/51	5	15	mEq/L	Latest pre-LT status snapshot; blood-draw date unavailable
Hospitalized before LT	REC_MED_COND (derived)	51/51	0	17	binary	Recipient medical condition immediately before LT; ICU or other hospitalization versus outpatient
BMI (audited; not modeled)	REC_BMI	49/51	2	16	kg/m2	Recipient preoperative clinical state
Adult Karnofsky (audited)	REC_FUNCTN_STAT (derived; instrument-specific)	25/51	26	6	score	Recipient pretransplant function; adult Karnofsky and pediatric Lansky kept separate
Pediatric Lansky (audited)	REC_FUNCTN_STAT (derived; instrument-specific)	16/51	35	6	score	Recipient pretransplant function; adult Karnofsky and pediatric Lansky kept separate
Laboratory MELD	CANHX_SRTR_LAB_MELD; M type	47/51	0 within M type	16	score	Latest strictly pre-LT status-history score
Laboratory PELD	CANHX_SRTR_LAB_MELD; P type	4/51	0 within P type	1	score	Latest strictly pre-LT status-history score; not pooled with MELD
ALT	REC_SGPT	27/51	24	14	U/L	17 strictly pre-LT; 10 same calendar date; observed in 2002-2014
AFP	CANHX_MPXCPT_AFP	13/51	38	3	ng/mL	Date-qualified pretransplant exception snapshot; not imputed
Current maximum diameter	CANHX_MPXCPT_HCC_TUMOR_SIZE_CM	11/51	40	3	cm	Within selected form; date-qualified imaging

Predictor	Source field	Observed / eligible	Missing or outside type	Recurrences among observed	Units	Timing/interpretation
Original maximum diameter	CANHX_MPXCPT_ORIG_HCC_TUMOR_SIZE	9/51	42	2	cm	Within selected form; historical original imaging

Counts refer to all 51 eligible recipients before excluding two with zero oncology observation. Recurrence counts cover complete follow-up. The status panel occurred 3 days before LT (IQR 1-16.5); its recording date is not a blood-draw date. Hospitalization combines intensive care and other hospitalization (9 patients) versus outpatient status (42). All 51 had associated-registration waiting time. Function instruments remain separate. All five status components were jointly available in 46 recipients with 15 recurrences; only sodium was imputed within training samples. AFP/count/vascular/extrahepatic/resection fields shared 13 recipients and were not used to fit a tumor-stage model. The model MELD/PELD score uses CANHX_SRTR_LAB_MELD and its type from the latest strictly prior status record. It is distinct from the CAN_LAST summary score reported in descriptive Table 1.

Adult Karnofsky, pediatric Lansky and legacy functional categories were not combined into one ordinal scale. BMI and other clinical fields were audited but were not added through outcome-based screening. No quantitative pretransplant platelet count, peripheral differential count, hemoglobin/hematocrit, LDH or CRP field was identified in the nine supplied liver candidate, recipient, status and exception families. The white-cell field concerning spontaneous bacterial peritonitis represented ascitic-fluid criteria, not a peripheral blood count. Post-transplant discharge laboratories were excluded.

Date-qualified recipient tumor snapshots were assessed separately from the candidate characterization in Table S8. AFP, tumor count, macrovascular and extrahepatic rule-out responses and resection candidacy were available in 13 recipients, current maximum diameter in 11 and original diameter in nine. Original application dates did not guarantee that form content was unchanged before transplantation; later status changes were retained in the audit. Sparse tumor variables were not imputed into the prediction models. A negative rule-out response does not prove invasion, and absence of a treatment date does not identify an untreated comparison group.

Exploratory prediction models and preprocessing

Model development proceeded in sequential locked extensions: demographic models after recurrence ascertainment; compact clinical and laboratory-component models after the clinical field audit; and an ALT substudy after its source/date review. Each extension fixed predictors, transformations, penalty, population and sensitivities before examining the new predictor-outcome associations. These were exploratory analyses added during manuscript development, not a prospectively registered prediction study. Table S13 lists the executed specifications.

Table S13. Locked exploratory prediction specifications

Model	Predictors and fixed transforms	Slope terms	Ridge penalty	Informative recipients	Scenarios	Resampling and seed
Demographic Fine-Gray	Age/10, male sex	2	None	49; pathology sensitivity 41	Primary recurrence date, first report date, discharge FLC	500 each; 20260903 + offsets 1-3
Demographic cause-specific ridge	Age/10, male sex	2	theta=1	Matched to each analysis	Same scenarios; clinical benchmark refitted in matched cohorts	500 each; same draws as matched model
Compact clinical ridge	Laboratory MELD, albumin, hospitalized, log(1+wait days)	4	theta=4	45 MELD; pathology sensitivity 38	Primary, first report date, discharge FLC	500 each; 20260915 + offsets 2,4,6
Laboratory-component ridge	log bilirubin, log INR, log creatinine, albumin, sodium, hospitalized, log(1+wait days)	7	theta=4	49; pathology sensitivity 41	Primary, first report date, discharge FLC	500 each; 20260915 + offsets 1,3,5
ALT + year ridge	log2 ALT, continuous transplant year	2	theta=4	27 / 17 / 26	Observed ALT; strictly pre-LT date; exclude ALT 0.67	500 each; 20260916 + offsets 1-3
Year-only ridge	Continuous transplant year	1	theta=4	Matched ALT subsets	Same ALT scenarios	Same draws as ALT + year
Null Aalen-Johansen	No predictors	0	Not applicable	Matched to each model	Training-sample event incidence	Same draws; constant prediction within evaluation sample

One year was secondary and three years primary. Every cause-specific model used a covariate-free training-sample competing-death hazard. No interactions, outcome-directed variable selection, penalty tuning or cutpoint search was performed. Natural logarithms were used except log2 ALT. Training medians preceded transformation; training mean and sample SD followed transformation. The empirical three-year threshold was refitted inside each training sample. Original demographic cause-specific models

used product-limit integration; expanded clinical models and matched demographic benchmarks report bounded exponential integration. ALT used bounded integration from its outset.

The initial demographic analysis used age per ten years and recorded male sex in a Fine-Gray subdistribution-hazard model and a cause-specific Cox model with fixed ridge penalty theta=1. An unpenalized cause-specific Cox model supplied descriptive associations. Fine-Gray fitting used cmprsk::crr with gtol=1e-6 and maxiter=10. Cause-specific models used Breslow ties. The clinical models used fixed ridge theta=4: MELD, albumin, hospitalization and log(1+waiting days), or natural-log bilirubin, natural-log INR, natural-log creatinine, albumin, sodium, hospitalization and log(1+waiting days). Predictor effects were unconstrained; no interactions, stepwise selection, penalty tuning or outcome-selected cutoffs were used.

For expanded clinical and ALT models, missing numeric values were replaced by the training-sample median on the raw scale, followed by the fixed transformation and training-sample mean/sample-standard-deviation standardization. The same fitted preprocessing was applied to held-out recipients. A predictor constant within a training sample had its coefficient set to zero; an entirely missing training predictor would cause an explicit failure. Only sodium required imputation in the selected clinical models. ALT was analyzed only when observed and was not imputed across its era-related absence. Demographic variables were complete and were standardized within each training sample.

Cause-specific recurrence models were paired with a covariate-free competing-death Cox hazard fitted within the same training sample; a sample without competing deaths used zero death hazard. This assumes a common competing-death hazard for the modeled recipients and was applied identically to matched comparators. Expanded models used coxph with iter.max=50, eps=1e-9 and scale=FALSE inside ridge after explicit standardization.

From cause-specific hazards to cumulative incidence

The original demographic analysis used product-limit cause-specific cumulative-incidence integration. During the expanded clinical analysis, some out-of-bag predictions produced fitted hazard increments greater than one; these were integration extrapolation failures rather than evidence of Cox nonconvergence. A numerical sensitivity retained all coefficients, penalties, training samples and predictors and replaced only integration with bounded exponential increments. These valid-probability results are the clinical estimates reported here.

For recurrence and competing-death increments hR and hD at each shared event-time interval, H=hR+hD. Recurrence probability was incremented by Sprev x [1-exp(-H)] x hR/H and event-free survival updated to Sprev x exp(-H), with zero increment when H=0. The corresponding death increment uses hD/H. This conserves total probability and remains bounded for large increments. Independent checks covered zero and large increments, probability-mass conservation and the constant-hazard case. No invalid probability was clipped or discarded in the bounded analysis. The ALT substudy used this integration from the outset.

Bootstrap validation and performance

Each scenario used 500 patient-level bootstrap samples of the informative cohort, drawn with replacement at the original cohort size. Recipients absent from a training sample were evaluated out of bag (OOB).

Model fitting, imputation, scaling, baseline hazards and the three-year median risk threshold were repeated inside the training sample. Compact/component models were compared with a demographic ridge model and an Aalen-Johansen null on the same draw and held-out recipients. ALT plus calendar year was paired with calendar year alone and the same null. Comparisons were made within matched populations; MELD-only, all-recipient and historical ALT cohorts were not ranked against one another by absolute AUC.

Performance was computed within each held-out sample and then averaged across evaluable samples. Cumulative/dynamic AUC treated recurrence by the horizon as a case and either survival without recurrence beyond the horizon or competing death by the horizon as a control. Tied predicted risks received half credit. Inverse probability of censoring weights used a Kaplan-Meier censoring estimate in the evaluation sample: 1/G(t-) for observed events and 1/G(horizon) for recipients observed beyond the horizon. Brier loss was weighted squared error summed over recipients and divided by evaluation-sample size; recipients censored before the horizon contributed zero weight. AUC was undefined without both cases and controls. Undefined metrics were counted separately from model or probability failures.

The null predicted each training sample's Aalen-Johansen recurrence probability for every held-out recipient, so its within-sample AUC was 0.5. Brier improvement is null or comparator Brier minus model Brier; positive values favor the model. Apparent full-cohort AUC is shown separately. Bootstrap 2.5th-97.5th percentiles describe the resampling distribution, not confidence intervals for external performance. Averaging repeated held-out predictions per recipient was used only for secondary descriptive calibration, not the principal discrimination estimate.

Table S14 reports the original demographic validation, and Figure S2 distinguishes apparent grouping from held-out prediction. In the primary demographic association model, the hazard ratio was 0.93 per decade of age (95% CI 0.67-1.30) and 0.51 for male sex (0.19-1.38); these associations did not define selection criteria.

Table S14. Demographic model validation at one and three years

**Scenario**	**Model**	**n / events**	**Year**	**Apparent<br>AUC**	**Mean OOB AUC (2.5th-97.5th percentiles)**	**Mean OOB Brier**	**Paired null Brier**	**Mean Brier improvement (2.5th-97.5th percentiles)**
Primary	Demographic Fine-Gray	49/17	1y	0.571	0.472 (0.078 to 0.819)	0.1719	0.1603	-0.0116 (-0.0621 to 0.0110)
Primary	Demographic Fine-Gray	49/17	3y	0.627	0.538 (0.181 to 0.817)	0.2163	0.2057	-0.0106 (-0.0895 to 0.0252)
Primary	Demographic ridge	49/17	1y	0.571	0.461 (0.079 to 0.787)	0.1718	0.1604	-0.0114 (-0.0581 to 0.0102)
Primary	Demographic ridge	49/17	3y	0.627	0.528 (0.182 to 0.817)	0.2167	0.2060	-0.0107 (-0.0888 to 0.0227)

Scenario	Model	n / events	Year	Apparent<br>AUC	Mean OOB AUC (2.5th-97.5th percentiles)	Mean OOB Brier	Paired null Brier	Mean Brier improvement (2.5th-97.5th percentiles)
First report date	Demographic Fine-Gray	49/17	1y	0.429	0.371 (0.059 to 0.818)	0.1084	0.1025	-0.0059 (-0.0255 to 0.0038)
First report date	Demographic Fine-Gray	49/17	3y	0.606	0.503 (0.200 to 0.794)	0.2122	0.1996	-0.0126 (-0.0790 to 0.0216)
First report date	Demographic ridge	49/17	1y	0.429	0.369 (0.059 to 0.818)	0.1082	0.1025	-0.0057 (-0.0249 to 0.0031)
First report date	Demographic ridge	49/17	3y	0.606	0.492 (0.170 to 0.793)	0.2126	0.1996	-0.0131 (-0.0793 to 0.0198)
Discharge pathology FLC	Demographic Fine-Gray	41/14	1y	0.495	0.405 (0.000 to 0.812)	0.1437	0.1344	-0.0093 (-0.0471 to 0.0084)
Discharge pathology FLC	Demographic Fine-Gray	41/14	3y	0.615	0.466 (0.096 to 0.806)	0.2077	0.1927	-0.0150 (-0.0848 to 0.0145)
Discharge pathology FLC	Demographic ridge	41/14	1y	0.523	0.415 (0.034 to 0.845)	0.1433	0.1349	-0.0084 (-0.0433 to 0.0079)
Discharge pathology FLC	Demographic ridge	41/14	3y	0.622	0.460 (0.107 to 0.800)	0.2076	0.1929	-0.0148 (-0.0813 to 0.0132)

OOB = out of bag. Results are means of within-sample evaluations. Parentheses show resampling-distribution percentiles, not confidence intervals for external performance. Lower Brier loss is better; improvement = null minus model Brier. Total recurrences cover all follow-up. The null AUC is 0.5 in each evaluable OOB sample. Undefined AUC evaluations are enumerated in Table S17. Apparent performance uses the full informative cohort and is distinct from held-out performance. Fine-Gray fitting succeeded in 499/500 primary and pathology-supported resamples and 500/500 report-date resamples; original ridge fitting succeeded in all 500 per scenario.

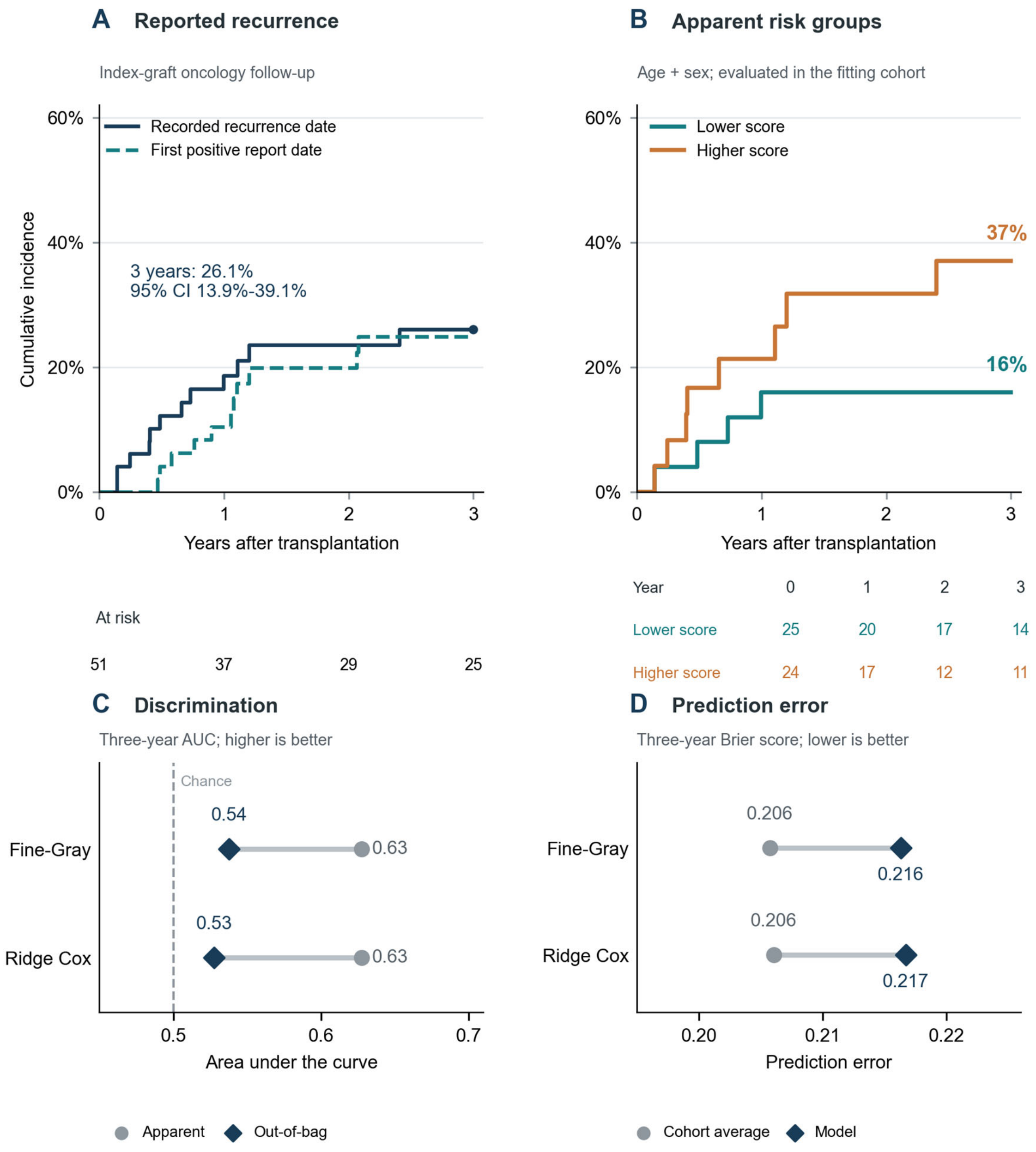

Figure S2. Center-reported recurrence and exploratory demographic risk models. (A) Aalen-Johansen recurrence estimates using the recorded recurrence date and first positive report date, with death without recorded recurrence competing; the primary three-year interval is a percentile interval from 5,000 patient bootstrap resamples. (B) Observed cumulative incidence in two groups divided at the median Fine-Gray predicted three-year risk in the same 49 recipients with post-LT oncology follow-up. This panel shows apparent fit, not validated risk classes. The two recipients without oncology follow-up were censored at day zero in panel A and excluded from modeling. (C-D) Mean within-out-of-bag-sample AUC and censoring-weighted competing-risk Brier score from 500 patient bootstrap samples. Each model and median threshold were refitted using training patients only. Fine-Gray and fixed-ridge cause-specific models used age and sex; the latter used a common competing-death hazard. The null comparator used each training sample's Aalen-Johansen risk. AUC values near 0.5 indicate little discrimination, and smaller Brier scores are better. Mean validation values are shown without interpreting bootstrap-distribution quantiles as confidence intervals.

Recurrence is registry reported, not centrally histologically adjudicated; these models do not establish transplant exclusion criteria.

Table S15 gives one- and three-year clinical validation with matched demographic and null comparators. The primary laboratory-component model had apparent three-year AUC 0.725 and mean held-out AUC 0.571; its mean Brier loss exceeded the null. The compact model similarly did not improve mean Brier performance. Report-date and pathology-supported sensitivities retained the same features and penalties.

Table S15. Clinical and laboratory models with matched demographic comparators

**Scenario**	**Model**	**n / events**	**Year**	**Apparent<br>AUC**	**Mean OOB AUC (2.5th-97.5th percentiles)**	**Mean OOB Brier**	**Paired null Brier**	**Mean Brier improvement (2.5th-97.5th percentiles)**
Primary, all score types	Matched demographic ridge	49/17	1y	0.571	0.472 (0.138 to 0.787)	0.1742	0.1637	-0.0105 (-0.0638 to 0.0093)
Primary, all score types	Matched demographic ridge	49/17	3y	0.627	0.533 (0.201 to 0.812)	0.2174	0.2078	-0.0096 (-0.0889 to 0.0253)
Primary, all score types	Laboratory components	49/17	1y	0.668	0.533 (0.182 to 0.923)	0.1779	0.1637	-0.0142 (-0.1108 to 0.0299)
Primary, all score types	Laboratory components	49/17	3y	0.725	0.571 (0.281 to 0.862)	0.2211	0.2078	-0.0134 (-0.1119 to 0.0444)
Primary, MELD type	Matched demographic ridge	45/16	1y	0.586	0.483 (0.068 to 0.901)	0.1642	0.1553	-0.0089 (-0.0511 to 0.0106)
Primary, MELD type	Matched demographic ridge	45/16	3y	0.635	0.532 (0.175 to 0.833)	0.2147	0.2063	-0.0084 (-0.0714 to 0.0232)
Primary, MELD type	Compact clinical	45/16	1y	0.585	0.441 (0.098 to 0.773)	0.1730	0.1553	-0.0176 (-0.0827 to 0.0116)
Primary, MELD type	Compact clinical	45/16	3y	0.641	0.507 (0.192 to 0.833)	0.2227	0.2063	-0.0164 (-0.1021 to 0.0305)
First report, all score types	Matched demographic ridge	49/17	1y	0.429	0.372 (0.056 to 0.865)	0.1021	0.0965	-0.0055 (-0.0246 to 0.0034)
First report, all score types	Matched demographic ridge	49/17	3y	0.606	0.509 (0.183 to 0.802)	0.2083	0.1981	-0.0101 (-0.0725 to 0.0178)

Scenario	Model	n / events	Year	Apparent<br>AUC	Mean OOB AUC (2.5th-97.5th percentiles)	Mean OOB Brier	Paired null Brier	Mean Brier improvement (2.5th-97.5th percentiles)
First report, all score types	Laboratory components	49/17	1y	0.728	0.587 (0.143 to 1.000)	0.1033	0.0965	-0.0068 (-0.0603 to 0.0225)
First report, all score types	Laboratory components	49/17	3y	0.729	0.585 (0.197 to 0.903)	0.2122	0.1981	-0.0141 (-0.1151 to 0.0518)
First report, MELD type	Matched demographic ridge	45/16	1y	0.432	0.353 (0.000 to 0.812)	0.1120	0.1055	-0.0065 (-0.0261 to 0.0045)
First report, MELD type	Matched demographic ridge	45/16	3y	0.616	0.499 (0.155 to 0.838)	0.2066	0.1963	-0.0104 (-0.0800 to 0.0219)
First report, MELD type	Compact clinical	45/16	1y	0.675	0.492 (0.000 to 0.923)	0.1109	0.1055	-0.0055 (-0.0349 to 0.0118)
First report, MELD type	Compact clinical	45/16	3y	0.688	0.559 (0.237 to 0.862)	0.2052	0.1963	-0.0090 (-0.0852 to 0.0356)
Discharge FLC, all score types	Matched demographic ridge	41/14	1y	0.523	0.420 (0.000 to 0.904)	0.1428	0.1352	-0.0076 (-0.0478 to 0.0076)
Discharge FLC, all score types	Matched demographic ridge	41/14	3y	0.622	0.467 (0.101 to 0.815)	0.2094	0.1965	-0.0129 (-0.0783 to 0.0188)
Discharge FLC, all score types	Laboratory components	41/14	1y	0.624	0.463 (0.068 to 1.000)	0.1520	0.1352	-0.0168 (-0.1498 to 0.0336)
Discharge FLC, all score types	Laboratory components	41/14	3y	0.692	0.523 (0.094 to 1.000)	0.2064	0.1965	-0.0098 (-0.1014 to 0.0654)

Scenario	Model	n / events	Year	Apparent<br>AUC	Mean OOB AUC (2.5th-97.5th percentiles)	Mean OOB Brier	Paired null Brier	Mean Brier improvement (2.5th-97.5th percentiles)
Discharge FLC, MELD type	Matched demographic ridge	38/13	1y	0.522	0.421 (0.000 to 0.915)	0.1289	0.1224	-0.0065 (-0.0375 to 0.0076)
Discharge FLC, MELD type	Matched demographic ridge	38/13	3y	0.606	0.474 (0.077 to 0.833)	0.2021	0.1901	-0.0120 (-0.0862 to 0.0166)
Discharge FLC, MELD type	Compact clinical	38/13	1y	0.575	0.404 (0.000 to 0.810)	0.1474	0.1224	-0.0250 (-0.2145 to 0.0092)
Discharge FLC, MELD type	Compact clinical	38/13	3y	0.625	0.469 (0.100 to 0.878)	0.2074	0.1901	-0.0173 (-0.1225 to 0.0480)

OOB = out of bag. Results are means of within-sample evaluations. Parentheses show resampling-distribution percentiles, not confidence intervals for external performance. Lower Brier loss is better; improvement = null minus model Brier. Total recurrences cover all follow-up. The null AUC is 0.5 in each evaluable OOB sample. Undefined AUC evaluations are enumerated in Table S17. Apparent performance uses the full informative cohort and is distinct from held-out performance. Every model has 500 valid bounded probability/Brier evaluations per scenario. Clinical and demographic rows within a scenario use identical resamples and populations. Absolute results from different populations are not direct model comparisons. Primary, all score types: three-year clinical-minus-demographic AUC 0.039 (-0.333 to 0.429); demographic-minus-clinical Brier -0.0037 (-0.1059 to 0.0920) (paired means and resampling percentiles). Primary, MELD type: three-year clinical-minus-demographic AUC -0.024 (-0.432 to 0.392); demographic-minus-clinical Brier -0.0080 (-0.0872 to 0.0725) (paired means and resampling percentiles). First report, all score types: three-year clinical-minus-demographic AUC 0.077 (-0.315 to 0.484); demographic-minus-clinical Brier -0.0040 (-0.1021 to 0.0771) (paired means and resampling percentiles). First report, MELD type: three-year clinical-minus-demographic AUC 0.060 (-0.324 to 0.518); demographic-minus-clinical Brier 0.0014 (-0.0706 to 0.0705) (paired means and resampling percentiles). Discharge FLC, all score types: three-year clinical-minus-demographic AUC 0.055 (-0.505 to 0.597); demographic-minus-clinical Brier 0.0031 (-0.0959 to 0.0942) (paired means and resampling percentiles). Discharge FLC, MELD type: three-year clinical-minus-demographic AUC -0.004 (-0.500 to 0.500); demographic-minus-clinical Brier -0.0053 (-0.1193 to 0.0813) (paired means and resampling percentiles).

Historical ALT substudy

REC_SGPT is a distinct pretransplant ALT field in U/L, supported by the dated pretransplant laboratory section of the official liver registration instructions. It was populated in 27 recipients, all transplanted in

2002-2014, including 14 with reported recurrence. Seventeen measurements preceded the transplant date; ten were on the same calendar date, with clock-time ordering unavailable. ALT was absent in 24 recipients, including all 23 transplanted in 2017-2025. This availability defined a historical selected substudy.

The locked ALT model used log2(ALT) and continuous transplant calendar year with ridge theta=4, compared with year alone at the same penalty and a training-cohort Aalen-Johansen null. Separate 500-sample validations used all 27 observed ALT values, the 17 strictly pretransplant measurements and 26 recipients after excluding an unresolved raw ALT value of 0.67 U/L. No model direction was reversed after seeing held-out AUC below 0.5. A separate unpenalized cause-specific Cox model with Breslow ties estimated the ALT hazard ratio per doubling adjusted for calendar year and its Wald 95% interval.

Table S16 reports prediction and descriptive association results. Mean three-year held-out AUC was 0.381, 0.383 and 0.463 across the three scenarios; Brier performance was worse than both comparators. Calendar-year adjustment does not supply validation in an era without ALT measurements.

Table S16. Historical ALT prediction and descriptive association analyses

Scenario	Model	n / events	Year	Apparent<br>AUC	Mean OOB AUC (2.5th-97.5th percentiles)	Mean OOB Brier	Paired null Brier	Mean Brier improvement (2.5th-97.5th percentiles)
Historical observed ALT	Year alone	27/14	1y	0.630	0.449 (0.003 to 0.875)	0.2257	0.2189	-0.0068 (-0.0481 to 0.0169)
Historical observed ALT	Year alone	27/14	3y	0.664	0.446 (0.026 to 0.833)	0.2771	0.2666	-0.0106 (-0.0748 to 0.0271)
Historical observed ALT	ALT + year	27/14	1y	0.611	0.357 (0.000 to 0.800)	0.2398	0.2189	-0.0209 (-0.0843 to 0.0193)
Historical observed ALT	ALT + year	27/14	3y	0.569	0.381 (0.000 to 0.771)	0.2991	0.2666	-0.0325 (-0.1150 to 0.0249)
Strictly pre-LT date	Year alone	17/9	1y	0.508	0.454 (0.000 to 1.000)	0.2490	0.2390	-0.0100 (-0.0808 to 0.0317)
Strictly pre-LT date	Year alone	17/9	3y	0.471	0.406 (0.000 to 1.000)	0.3137	0.2894	-0.0243 (-0.1450 to 0.0323)
Strictly pre-LT date	ALT + year	17/9	1y	0.650	0.454 (0.000 to 1.000)	0.2556	0.2390	-0.0166 (-0.1400 to 0.0690)
Strictly pre-LT date	ALT + year	17/9	3y	0.557	0.383 (0.000 to 1.000)	0.3349	0.2894	-0.0455 (-0.1966 to 0.0463)

Scenario	Model	n / events	Year	Apparent<br>AUC	Mean OOB AUC (2.5th-97.5th percentiles)	Mean OOB Brier	Paired null Brier	Mean Brier improvement (2.5th-97.5th percentiles)
Exclude ALT 0.67	Year alone	26/13	1y	0.664	0.470 (0.000 to 0.946)	0.2063	0.2020	-0.0043 (-0.0505 to 0.0189)
Exclude ALT 0.67	Year alone	26/13	3y	0.688	0.477 (0.044 to 0.962)	0.2666	0.2579	-0.0087 (-0.0899 to 0.0355)
Exclude ALT 0.67	ALT + year	26/13	1y	0.455	0.389 (0.000 to 1.000)	0.2156	0.2020	-0.0137 (-0.0891 to 0.0365)
Exclude ALT 0.67	ALT + year	26/13	3y	0.599	0.463 (0.000 to 1.000)	0.2760	0.2579	-0.0180 (-0.1404 to 0.0518)

OOB = out of bag. Results are means of within-sample evaluations. Parentheses show resampling-distribution percentiles, not confidence intervals for external performance. Lower Brier loss is better; improvement = null minus model Brier. Total recurrences cover all follow-up. The null AUC is 0.5 in each evaluable OOB sample. Undefined AUC evaluations are enumerated in Table S17. Apparent performance uses the full informative cohort and is distinct from held-out performance. Historical observed ALT: descriptive year-adjusted Cox HR per ALT doubling 0.94 (95% CI 0.61-1.45); three-year Brier improvement over year alone -0.0220 (-0.0926 to 0.0103). Strictly pre-LT date: descriptive year-adjusted Cox HR per ALT doubling 0.82 (95% CI 0.58-1.18); three-year Brier improvement over year alone -0.0211 (-0.1487 to 0.0450). Exclude ALT 0.67: descriptive year-adjusted Cox HR per ALT doubling 1.54 (95% CI 0.81-2.92); three-year Brier improvement over year alone -0.0094 (-0.1257 to 0.0419). All 500 draws produced valid probabilities/Brier scores in each scenario. Three-year recurrence counts were 10/27, 7/17 and 9/26, respectively. No ALT values were imputed; all observed values arose in 2002-2014.

Relative group reproducibility and numerical verification

Within every training sample, predicted three-year recurrence probability strictly above the training median defined the higher relative group; ties were assigned lower. Each recipient's higher-group assignment probability used only their OOB predictions. Stable lower or higher assignment required probability at most 0.20 or at least 0.80, respectively; intermediate assignments were variable. These measure classification consistency, rather than validated low- and high-risk transplant categories. Apparent group Aalen-Johansen differences and pooled OOB calibration were descriptive. Each recurrence was also omitted once to assess coefficient, predicted-risk and assignment influence.

Table S17 reports successful probability evaluations, undefined AUC counts, the retained product-limit integration failures, assignment stability and single-event influence. Figure S3 summarizes primary clinical performance and assignment reproducibility. All 3,000 expanded clinical draws and all 1,500 ALT draws produced valid bounded predictions. The original Fine-Gray models had one nonconvergent

bootstrap fit in the primary scenario and one in the pathology-supported scenario; the remaining demographic fits completed.

Table S17. Resampling validity, relative assignment stability and single-event influence

Scenario	Model	Valid fit/probability draws	Evaluable AUC draws, 1/3 years	Original PL integration failures	Stable lower / higher / variable, n	Maximum risk change after one event omitted, percentage points	Maximum apparent assignments changed
Primary	Demographic Fine-Gray	499/500	486/496	Not applicable	16/17/16	6.0	1
Primary	Demographic ridge	500/500	487/497	Not applicable	16/17/16	5.6	1
First report date	Demographic Fine-Gray	500/500	460/497	Not applicable	15/17/17	5.4	1
First report date	Demographic ridge	500/500	460/497	Not applicable	14/14/21	5.1	1
Discharge pathology FLC	Demographic Fine-Gray	499/500	466/494	Not applicable	4/6/31	5.5	8
Discharge pathology FLC	Demographic ridge	500/500	467/495	Not applicable	2/4/35	5.3	10
Primary, all score types	Laboratory components	500/500	490/498	28	11/13/25	19.1	5
Primary, MELD type	Compact clinical	500/500	490/495	1	10/13/22	17.1	6
First report, all score types	Laboratory components	500/500	449/498	35	13/15/21	18.8	4
First report, MELD type	Compact clinical	500/500	444/497	1	11/13/21	16.3	6
Discharge FLC, all score types	Laboratory components	500/500	464/491	76	7/10/24	22.7	5
Discharge FLC, MELD type	Compact clinical	500/500	451/493	66	6/8/24	32.4	8
Historical observed ALT	ALT + year	500/500	485/499	Not applicable	1/0/26	38.3	18
Strictly pre-LT date	ALT + year	500/500	453/484	Not applicable	2/1/14	44.0	6

Scenario	Model	Valid fit/probability draws	Evaluable AUC draws, 1/3 years	Original PL integration failures	Stable lower / higher / variable, n	Maximum risk change after one event omitted, percentage points	Maximum apparent assignments changed
Exclude ALT 0.67	ALT + year	500/500	467/493	Not applicable	3/4/19	32.9	8

Each analysis attempted 500 resamples. Missing AUCs indicate an evaluation sample without cases or controls, rather than a failed fit when predictions were valid. Original Fine-Gray exceptions were convergence failures. PL denotes earlier product-limit integration; the displayed bounded clinical results retain all 500 draws without those probability failures. Matched clinical demographic and ALT year-only comparators also had 500/500 valid bounded probability draws. Their evaluable AUC counts equal those of the paired model in Tables S15–S16. Stable assignments use only held-out predictions and require at least 80% lower or higher assignments against each training median. The remainder are variable. These are consistency labels rather than clinical risk probabilities. Influence values are maxima across leave-one-recurrence-out fits, not a validation metric.

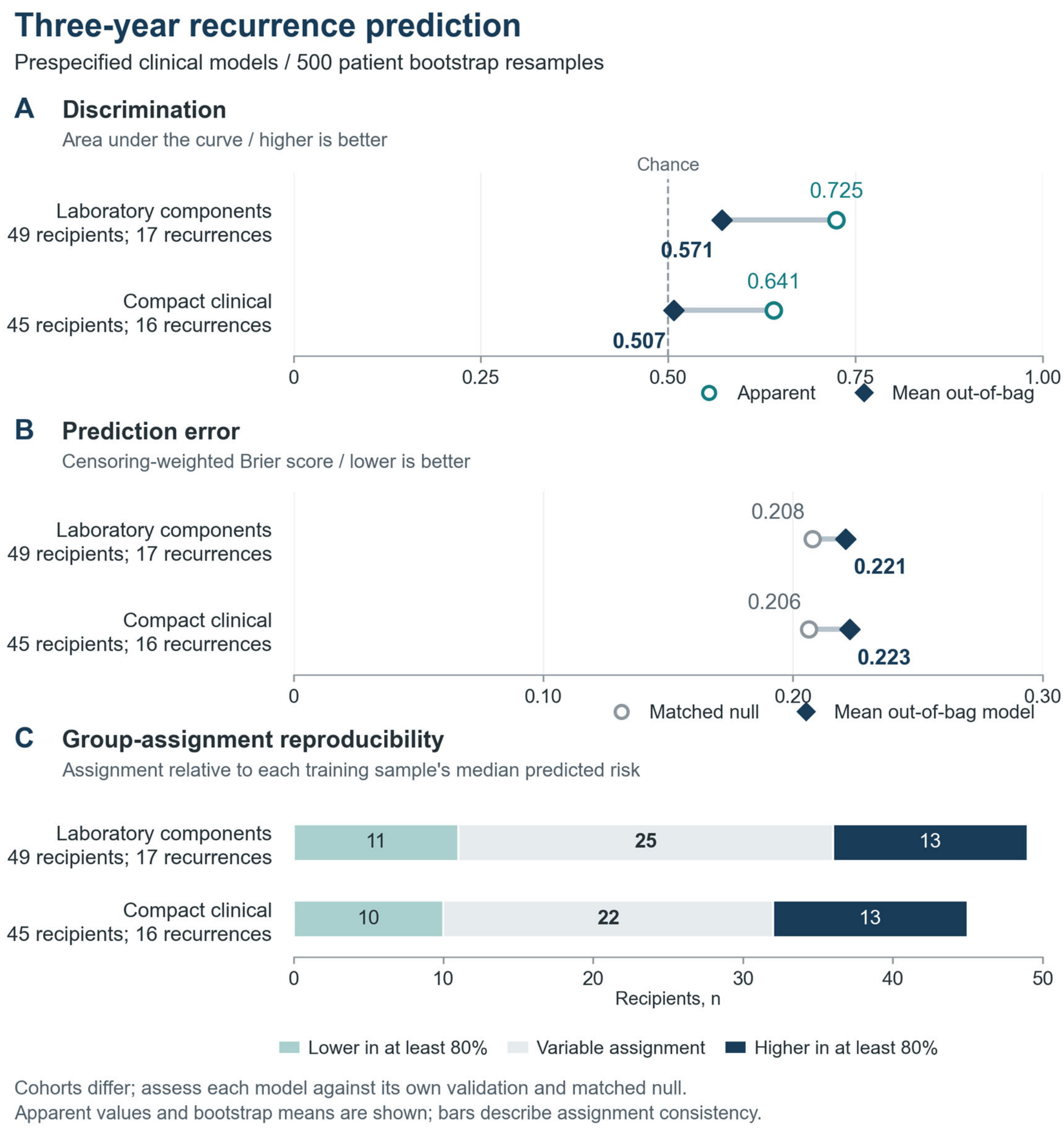

Figure S3. Internal validation of clinical recurrence prediction after liver transplantation for fibrolamellar carcinoma. The laboratory-component model included 49 recipients (17 reported recurrences); the compact MELD model included 45 (16). (A) Apparent and mean out-of-bag three-year AUC; the dashed line denotes chance ranking (0.5). (B) Mean censoring-weighted competing-risk Brier loss versus each model's own training-cohort Aalen-Johansen null, evaluated in the same held-out recipients. Lower loss is better. (C) Relative-group assignment reproducibility. Each of 500 bootstrap draws refitted preprocessing, models and the training-only median predicted three-year risk threshold. Assignments were stable when at least 80% of held-out predictions placed the recipient in that group; others were variable. Numbers show recipient counts within each cohort. Bounded competing-hazard integration produced 500 valid probability/Brier evaluations per model; AUC was evaluable in 498 and 495 draws, respectively. Different populations prevent direct comparison of absolute AUCs. Relative groups describe assignment consistency, not validated clinical risk classes. The endpoint is reported pretransplant-tumor recurrence in FLC recipients, without central histologic adjudication.

Independent review verified raw source/date mappings, fixed outcome vectors, training-only preprocessing, resample exclusion and selected held-out AUC/Brier calculations. The bounded clinical sensitivity reused identical bootstrap draws and fitted coefficients. The ALT audit checked 4,500 training-feature calculations and independently reproduced the three descriptive Cox models. Analyses used R 4.5.1, survival 3.8-3 and cmprsk for Fine-Gray estimation. Base seeds were 20260903 for original demographic models, 20260915 for expanded clinical models and 20260916 for ALT, with scenario offsets specified in Table S13. Executed scripts and aggregate outputs were retained with content hashes.

Liver explant analysis

A descriptive analysis used the registry liver explant file (LIVER_EXPLANT_DATA), which records pathological findings from the explanted liver, with first native-liver recipients carrying DIAG=4402 and entry February 27, 2002-December 31, 2025. Linkage used PT_CODE, TRR_ID_CODE and TRR_FOL_ID_CODE within that file. This cohort contained 44 recipients; ten had an index-graft explant form and eight had complete core fields. The explant file carries its own encrypted transplant identifier, which is not the numeric TRR_ID of the primary analysis files, so the explant records could not be merged with the primary cohorts. The explant analysis is a second pass over the same registry rather than an independent validation sample, and the two were not pooled.

The registry file documentation specifies explant collection for transplants beginning April 12, 2012 with an approved HCC exception at transplantation. Eight recipients had complete vascular invasion, regional node, extrahepatic spread, tumor number, differentiation and first-lesion size fields; satellites and first-lesion location/necrosis were also recorded in those eight. The explant file schema contained no pathology narrative, report identifier, specimen date, margins, examined/positive node counts or fusion results. Another graft in the same-person search supplied no additional explant form and did not replace the index native explant. Table S18 describes capture and field meaning.

Table S18. Capture and meaning of structured explant fields

Measure or field	Capture	Source/definition
First-native FLC cohort in the explant file	44	DIAG4402; within-file identifiers
Index explant form	10/44	HCC-exception ascertainment beginning 2012-04-12
Complete core fields	8/10 forms	Vascular, nodes, extrahepatic, tumor number, differentiation, first size
Index oncology forms	43/44 recipients; 323 forms	Linked to index graft; not generic vital follow-up
Vascular Invasion	8/10 forms	VASCULAR_INVASION
Lymph Node Involvement	8/10 forms	LYMPHNODEINVOLVEMENT
Other Extrahepatic Spread	8/10 forms	EXTRAHEPATICSPREAD
Worst Tumor Differentiation	8/10 forms	WORST_TUMOR_DIFF
Satellite Lesions	8/10 forms	SATELLITELESIONS
Number of Tumors	8/10 forms	NUMBER_TUMORS

Measure or field	Capture	Source/definition
Tumor #1 - Size	8/10 forms	TUMOR_SIZE1
Tumor #1 - Necrosis	8/10 forms	TUMOR_NECR1
Tumor #1 - Location	8/10 forms	TUMOR_LOC1
Evidence of HCC found in explant	9/10 forms	EVIDENCEOFHCC
Pre-transplant treatment for HCC?	9/10 forms	TREATMENTFORHCC
Narrative, margins, nodes examined/positive, molecular fusion	Not present in supplied explant schema	No report identifier or specimen-date field
Additional explant on another same-person graft	0	One other graft found; index explant not replaced

The explant cohort is drawn from the same registry as the primary cohorts; because the transplant identifier is separately encrypted, no record-level matching, pooling or independent validation was inferred. Eight recipients had complete core fields; this does not mean eight required fields. The explant form is postoperative and conditionally collected. Literal vascular "None" is negative; blank is missing. Unused lesion slots are structural. Size denotes largest dimension in centimeters in each recorded lesion. EVIDENCEOFHCC=N on the HCC form does not reverse FLC eligibility.

Blank values remained missing. Literal vascular invasion "None" represented explicit absence; unknown categories were not recoded negative. A tumor count of >5 remained an open category. Unused lesion slots were structural, and active recorded lesions were analyzed separately. The maximum recorded diameter used the largest dimension in centimeters among active lesion slots, not an assumption that slot 1 was necessarily the largest. Necrosis was categorical: none, incomplete (viable tumor remains), or complete (no viable tumor). EVIDENCEOFHCC=N on an HCC-specific form did not erase the FLC cohort diagnosis.

Explant-file oncology follow-up and descriptive pathology comparisons

Before this focused outcome review, adverse pathological extent was defined as any microvascular or macrovascular invasion, regional node involvement or extrahepatic spread. The comparator required explicit absence of all three; incomplete records without an affirmative adverse feature were unclassified. Four recipients had adverse extent, four had all three features explicitly absent and two were unclassified. No regression, significance test, weighted score, diameter cutoff or optimized grouping was fitted.

Recurrence required MALIG_PRETX_TUMOR=Y with a valid RECUR_DATE on or after index transplantation and no later than the next graft, administrative cutoff or verified death. Index-graft oncology and parent general follow-up dates were reconciled. An explicit negative assessment was MALIG=N or MALIG_PRETX_TUMOR=N before any first recurrence. Follow-up ended at the last such assessment, not generic vital contact. Competing death required oncology observation extending to death without an earlier censoring gap. One- and three-year horizons were 365.25 and 1095.75 days. A recorded negative through a horizon is an observed assessment, not independent confirmation of disease-free status.

Two reported events occurred among the four recipients with adverse extent and none among those with all three features explicitly absent. At three years, the adverse group comprised one recurrence, two recipients with a prior negative assessment covering the horizon and one with unknown status; the

explicit-absence group comprised three negative-covered recipients and one unknown. No competing deaths were counted at these horizons. Table S19 retains horizon-specific unknown status and first-positive-report timing.

Table S19. Pathological extent and horizon-specific oncology observation

Extent category	n	Total reported recurrences	Horizon	Recurrence by horizon	Negative assessment covers horizon	Unknown at horizon	First positive report by horizon
All three explicitly absent	4	0	1 year	0	4	0	0
All three explicitly absent	4	0	3 year	0	3	1	0
Adverse extent recorded	4	2	1 year	1	3	0	0
Adverse extent recorded	4	2	3 year	1	2	1	1
Unclassified	2	0	1 year	0	1	1	0
Unclassified	2	0	3 year	0	1	1	0

Adverse extent means any microvascular or macrovascular invasion, positive regional nodes or extrahepatic spread. Explicit absence requires all three negative. Missing records without an affirmative adverse feature are unclassified. Horizon lengths are 365.25 and 1095.75 days. A negative assessment must precede an established first recurrence; subsequent negative forms cannot restore recurrence freedom. No competing deaths were counted at these horizons. The two reported events occurred at 52 and 1,499 days; first positive reports followed by 706 and 99 days. Two intervening negative forms affected one event. The report-date change at one year does not create verified recurrence freedom. Among recipients without reported recurrence, last-negative follow-up was 969-4,428 days (median 3,615.5) in the explicit-absence group and 2,977-4,655 days in the two adverse-extent non-event recipients. Recorded negative assessments are not independent disease-free adjudication.

The two events were recorded at 52 and 1,499 days, with first-positive-report delays of 706 and 99 days. Two intervening negative forms preceded the early first positive report. Under report-date timing, the number of adverse-group events by one year changed from one to zero; by three years it remained one. Negative forms after the first recurrence were not used to restore recurrence freedom. The four explicit-absence recipients without an event had last-negative follow-up of 969-4,428 days (median 3,615.5); three reached three years. The two adverse-extent recipients without an event had 2,977 and 4,655 days of negative follow-up.

The event categories were one hepatic/biliary tumor and one other tumor. Their existing pretransplant diagnostic strings did not affirmatively identify another primary malignancy, but recurrence identity remained unadjudicated. There were no same-row MALIG=N with MALIG_PRETX_TUMOR=Y

contradictions among the ten explant recipients. Two recurrence dates later than their parent assessment occurred in the wider 44-recipient cohort, not in these ten.

Table S20 describes each pathological feature without converting the extent pattern into a validated score. Both event-bearing explants lacked recorded vascular invasion and satellites; one had nodal involvement and the other extrahepatic spread. Maximum recorded diameters overlapped substantially between event and non-event recipients. One recipient had complete necrosis in every recorded lesion and no reported recurrence; seven had viable tumor in at least one lesion, including both events. These postoperative observations support further pathology-linked study and cannot themselves establish pretransplant eligibility.

Table S20. Individual explant features and reported recurrence

Feature	Category	Recipients	Reported recurrences
Vascular invasion	None	6	2
Vascular invasion	Macrovascular	1	0
Vascular invasion	Microvascular	1	0
Vascular invasion	Missing	2	0
Regional nodes	Absent	6	1
Regional nodes	Present	2	1
Regional nodes	Missing	2	0
Extrahepatic spread	Absent	7	1
Extrahepatic spread	Present	1	1
Extrahepatic spread	Missing	2	0
Satellite lesions	Absent	5	2
Satellite lesions	Present	3	0
Satellite lesions	Missing	2	0
Differentiation	Well	3	1
Differentiation	Moderate	5	1
Differentiation	Missing	2	0
Tumor multiplicity	Multiple	4	1
Tumor multiplicity	Single	4	1
Tumor multiplicity	Unknown	2	0
Necrosis in all active recorded lesions	Complete	1	0
Viability in at least one recorded lesion	None or incomplete necrosis	7	2

Counts are descriptive and categories overlap across features. Two of ten forms had incomplete core fields. Both events lacked vascular invasion and satellite lesions; event-bearing recipients instead had nodal involvement or extrahepatic spread. The eight core recipients contributed 20 active recorded lesion

slots. Necrosis was categorical rather than a percentage or treatment-response score. No p-values, regression model, tumor-size threshold or eligibility rule was fitted. Maximum recorded explant diameter among event recipients (n=2): median 5.05 cm, range 0.6-9.5 cm. Maximum recorded explant diameter among non-event recipients (n=6): median 6.70 cm, range 1.0-14.5 cm.

External molecular evidence and reproducible research priorities

Table S21 summarizes three directly reviewed external molecular studies discussed in the manuscript. These gene-expression, methylation and circulating-DNA observations are literature evidence, not measurements in either registry cohort. The table distinguishes resection prognosis, disease monitoring and transplantation representation rather than importing external markers into the registry models.

Table S21. External molecular candidates: population, endpoint and transplantation applicability

Study	Assay or candidate	Studied population	Transplant representation	Actual evidence	Applicability/data boundary
Cornella et al., 2015 [37]	Gene-expression mortality index: PEAR1, KRTAP, KLRD1, OSBPL8, RPL32, SLC26A11, RGS11, RAPGEF1	29 resected for training survival; 26 for recurrence; 22 French resected survival validation	Four transplants in broader clinical series; explicitly excluded from prognostic analyses	Resection survival signature; recurrence analysis in training cohort. Upper-quartile thresholds rederived by cohort/outcome (21, 25, 8).	GSE57727 expression is public; metadata lack transplant/outcome variables. Not a post-LT validated panel.
Malouf et al., 2015 [38]	LINE-1 global methylation within tumor methylome study	20 resected pure FLC; low LINE-1 group 6 versus other 14	No validated transplant-specific prognostic analysis	Lower LINE-1 associated with shorter recurrence-free survival; exploratory threshold based on mean minus SEM. Diagnostic methylation validation differs from prognostic validation.	Testable epigenetic candidate; no adjusted/external post-LT recurrence classifier.
McKenna et al., 2023 [39]	Tumor-informed circulating tumor DNA; Signatera	51 patients; 226 samples; 92% stage III/IV	No transplant-specific recurrence prediction validation reported	Serial monitoring with imaging in a conference abstract; not a transplant selection study.	Conference evidence. No assay-target identity or performance denominator beyond the source was inferred.

Literature only: none of these assays or genes was measured in this registry analysis. Reference numbers correspond to the integrated manuscript. Cornella and Malouf DOIs were verified through PubMed. McKenna is an ASCO conference abstract verified through the official meeting record and Google

Scholar, not a PubMed-indexed full paper. A prognostic association, diagnostic fusion or monitoring assay does not establish benefit from offering or withholding transplantation. KRTAP is the source label and is not assigned to an unreported family member.

The eight-gene mortality index reported by Cornella and colleagues contains PEAR1, KRTAP, KLRD1, OSBPL8, RPL32, SLC26A11, RGS11 and RAPGEF1. KRTAP is retained as published rather than assigned an unsupported family-member identifier. Although the broader clinical series included four transplant recipients, prognostic analyses explicitly excluded transplanted patients. Training recurrence and external resection survival analyses therefore do not validate post-transplant recurrence prediction. GSE57727 contains expression data but does not itself provide a public transplant/outcome-labelled validation cohort.

Prospective testing would require molecularly and pathologically characterized FLC with pretreatment imaging, stage, serial response and postoperative recurrence adjudication, followed by a separately validated model. A diagnostic driver fusion, an association with prognosis after resection or detection of circulating tumor DNA is not by itself evidence that withholding transplantation improves outcomes. No molecular eligibility rule was derived from the present analyses.

Supplementary References

Numbering follows the main manuscript.

37. Cornella, H.; Alsinet, C.; Sayols, S.; Zhang, Z.; Hao, K.; Cabellos, L.; Hoshida, Y.; Villanueva, A.; Thung, S.; Ward, S.C.; et al. Unique genomic profile of fibrolamellar hepatocellular carcinoma. *Gastroenterology* **2015**, *148*, 806–18.e10. https://doi.org/10.1053/j.gastro.2014.12.028

38. Malouf, G.G.; Tahara, T.; Paradis, V.; Fabre, M.; Guettier, C.; Yamazaki, J.; Long, H.; Lu, Y.; Raynal, N.J.; Jelinek, J.; et al. Methylome sequencing for fibrolamellar hepatocellular carcinoma depicts distinctive features. *Epigenetics* **2015**, *10*, 872–81. https://doi.org/10.1080/15592294.2015.1076955

39. McKenna, E.; Kent, P.; Tasse, J.C.; Schadde, E.; Fisher, O.M.; Humar, A.; Kato, T.; Ellison, J.; Yamashiro, D.; Cornelius, A. First report of tumor-informed circulating tumor DNA in fibrolamellar carcinoma in adolescents and young adults: 51 patients (abstract). *J. Clin. Oncol.* **2023**, *41 (16_suppl)*, 3034. https://doi.org/10.1200/JCO.2023.41.16_suppl.3034
